# Advances in large-scale electrophysiology with high-density microelectrode arrays

**DOI:** 10.1039/d5lc00058k

**Published:** 2025-08-28

**Authors:** Manuel Schröter, Fernando Cardes, Cat-Vu H. Bui, Lorenzo Davide Dodi, Tobias Gänswein, Julian Bartram, Lorenca Sadiraj, Philipp Hornauer, Sreedhar Kumar, Maria Pascual-Garcia, Andreas Hierlemann

**Affiliations:** a Department of Biosystems Science and Engineering, ETH Zürich 4056 Basel Switzerland manuel.schroeter@bsse.ethz.ch andreas.hierlemann@bsse.ethz.ch

## Abstract

A detailed functional characterization of electrogenic cells, such as neurons and cardiomyocytes, by means of high-density microelectrode arrays (HD-MEAs) has emerged as a powerful approach for inferring cellular phenotypes and elucidating fundamental mechanisms underlying cellular function. HD-MEAs have been applied across a range of disciplines, including neurodevelopmental research, stem cell biology, and pharmacology, and more recently in interdisciplinary work at the intersection of biomedical engineering, computer science, and artificial intelligence (AI). Innovations in chip design, fabrication, recording capabilities, and data processing have significantly advanced the functionality of HD-MEAs. Today's chips allow the study of cellular function across scales and at high throughput. They enable the analysis of multi-parametric functional phenotypes over extended time and facilitate monitoring the effects of targeted perturbations on cellular behavior. In this *Tutorial Review*, we will first survey the advances in HD-MEA design and their readout and stimulation capabilities. We will then abstract studies that used HD-MEAs in combination with other experimental techniques to probe biologically relevant cellular and subcellular features, with an emphasis on *in vitro* applications of HD-MEAs. Thereafter, we will cover analytical techniques that are essential for analyzing and characterizing HD-MEA data. Finally, we will address current limitations of HD-MEAs and discuss potential future developments.

## Abstract 2

This review provides an overview on advances in high-density microelectrode array (HD-MEA) technology and explores its application in probing electrogenic cell function at high spatiotemporal resolution and across scales.

## Graphical abstract

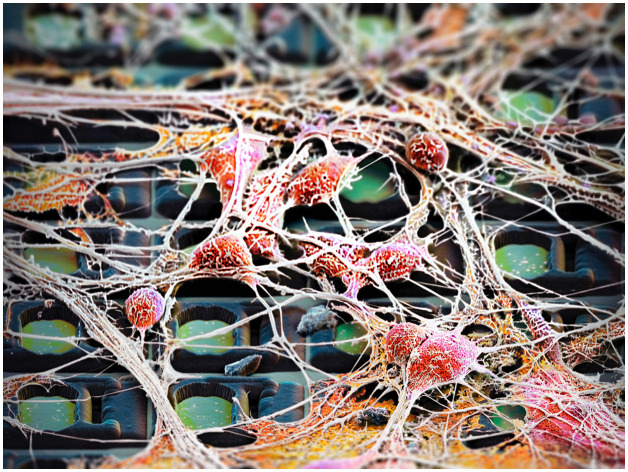

## 1. Introduction

Microelectrode arrays (MEAs) have gained significant attention in recent years, driven in part by exciting advances in brain–computer interface (BCI) technology and a dynamically evolving industry behind this trend.^1,2^ Equally important is an increasing body of basic neuroscience research that has employed MEAs to probe the functional properties of neurons in living animals^3^ and in *in vitro* model systems. The use of MEAs *in vitro* has been further accelerated by the rise of human stem cell-derived neuronal cultures, which allow researchers to recapitulate both physiological and disease-relevant states in the dish.^4–6^

Advanced high-density MEAs (HD-MEAs) represent a key enabling tool for both *in vivo* and *in vitro* research. They facilitate recordings across a wide range of spatial scales – from subcellular compartments and individual cells to entire intact networks – and across temporal scales, spanning microseconds to months.^7,8^ In addition, HD-MEAs allow efficient interaction with cells through targeted electrical stimulation.^9,10^ These capabilities render HD-MEAs highly valuable for advancing our understanding of fundamental electrophysiological mechanisms and for exploring biological systems in greater depth. HD-MEAs are also increasingly employed in translational applications, such as functional phenotyping of human cellular models and drug screening. Here, they provide insights that are often inaccessible through other characterization techniques, such as patch-clamp or calcium imaging.

Advances in microfabrication, and specifically complementary metal-oxide-semiconductor (CMOS) technology, enabled the miniaturization of key components of MEAs – such as the electrodes – and the integration of electronic components, including filters, amplifiers and analog-to-digital converters (ADCs) directly on the chip. The use of integrated electronics in HD-MEAs helped to overcome the “connectivity problem”^11^ of traditional low-density, passive MEA devices and to significantly enhance the overall number of electrodes, the array area, the spatial density of electrodes (>3000 per mm^2^), and the number of readout channels. Furthermore, the proximity of the microelectrodes to the integrated electronics improved the signal-to-noise ratio (SNR) by avoiding long signal paths, which would entail more parasitic capacitance leaks, resistive losses, and thermal noise proportional to the resistive losses.

A recent planar HD-MEA device,^12^ for example, featured a sensing area of 5.51 × 5.91 mm^2^ accommodating 236 880 electrodes (electrode size 11.22 × 11.22 μm^2^, with only 0.25 μm spacing between neighboring electrodes) and enabling the simultaneous readout of 33 840 channels at 70 kHz. Such devices offer unprecedented detail, enabling large-scale, high-density recordings across multiple spatial and temporal scales - from tracking local field potential (LFP) dynamics in specific layers of a thalamo-cortical slice to monitoring action potential (AP) propagation along the axonal arbors of individual neurons. As we will discuss here, such impressive recording capabilities have been even further augmented by adding other readout modalities to HD-MEAs, and by introducing innovative electrode designs for intracellular-like measurements at scale.^13^

There are a number of previous reviews covering developments in the MEA field.^14–22^ Obien *et al.*^14^ provided a comprehensive overview on advances in CMOS-based MEA technology at the time, while other reviews focused on advances in fabrication techniques for planar,^15,16,21^ three-dimensional (3D),^16–18^ or flexible MEAs^19,20^ and their suitability for next-generation neuronal interfaces.^17,22^ In this *Tutorial Review*, we will cover advancements in HD-MEA technology and cutting-edge applications within the past decade (2014–2024). In particular, we will focus on technological innovations that enabled these systems to become attractive platforms for multimodal investigations of cellular function in *in vitro* cultures and *ex vivo* tissue preparations. We expect that the presented technology and applications are not only relevant for future endeavors in basic and translational neuroscience, but will be used for advanced “electrical imaging” in many other fields of bioelectrical signaling and regulation – across health and disease.^23^

The structure of the review is as follows: after the introduction, section 2 focuses on recent advances in chip design and fabrication that provide the foundation for the advanced recording capabilities of today's HD-MEAs. Specifically, we will review technological innovations that have led to improved spatiotemporal sampling of bioelectric signals, higher numbers of readout- and stimulation sites, and new multimodal strategies to record previously inaccessible cellular signals. Section 3 will provide an overview on HD-MEA studies that applied HD-MEA systems in combination with other experimental tools to study cellular/network-level electrophysiology. In section 4, we will review innovative data analysis techniques for HD-MEA recordings. Finally, we conclude the review with a discussion on current limitations and potential future directions in this highly interdisciplinary field (section 5).

## 2. Advances in HD-MEA chip design and fabrication

The design of MEAs typically begins with defining the specifications for the target application. These include two primary aspects: the readout modalities and the electrode array connectivity. MEAs can be engineered for different purposes: while some designs prioritize multi-functionality, others are optimized for high throughput. Moreover, while certain designs focus on achieving high spatiotemporal resolution, other designs prioritize high adaptability to dynamic experimental conditions. These diverse design objectives have led to the development of a broad range of MEA architectures, each tailored to specific applications.

A central challenge in HD-MEA design is establishing connectivity between the densely packed electrodes and their associated readout circuits (*cf.* Box 1). In many designs, the circuits are positioned at the periphery outside the electrode array rather than directly underneath the electrodes, which adds complexity to establishing efficient and reliable connections. Effectively addressing this issue is critical for ensuring optimal recording performance and to prevent signal degradation.

**Box 1: Design of HD-MEAs**

The design of CMOS-based high-density microelectrode arrays (HD-MEAs) involves several critical trade-offs that must be carefully balanced to optimize performance, costs, and practical use. Key considerations include the choice of technology node, array density and size, channel count and signal quality, circuit integration, and data readout strategies.

**Technology node**

Smaller technology nodes (*e.g.*, 90 nm, 65 nm, 40 nm) enable advanced digital processing capabilities but come with higher fabrication costs and offer limited advantages for analog circuits. Conversely, older nodes (*e.g.*, 130 nm, 180 nm, 350 nm) are better suited for analog design but result in larger, less efficient digital circuits due to increased feature size.

**Number of channels and signal quality**

The chip real estate and power consumption of each readout circuit are directly tied to the desired signal quality (*e.g.*, noise level). As the number of channels increases, integrating numerous readout circuits becomes more challenging. High channel counts can result in excessive power consumption, which may lead to heat dissipation issues, as well as increased chip area, ultimately raising fabrication costs.

**On-chip *vs.* off-chip circuitry**

Integrating all readout components – including amplifiers, filters, and ADCs – directly on-chip preserves signal integrity but is resource-intensive. Alternatively, placing some circuits off-chip reduces on-chip resource demands but may include the risk of degrading signal quality due to the transmission of low-level analog signals through external connections.

**Density *vs.* array size**

For a given number of electrodes, electrode density and array size are inversely related. Designers must balance the trade-off between a smaller, high-density array that offers greater spatial resolution over a limited area, and a larger, lower-density array that sacrifices resolution in favor of broader coverage.

**Full-frame *vs.* partial readout**

In very large arrays with tens of thousands of electrodes, reading out all electrodes (full-frame readout) generates massive data volumes and poses significant circuit-design challenges. Alternatively, focusing on a subset of electrodes (partial readout) reduces data volume and system complexity but may risk missing important information from unmonitored array areas.

**Fig. 1:**
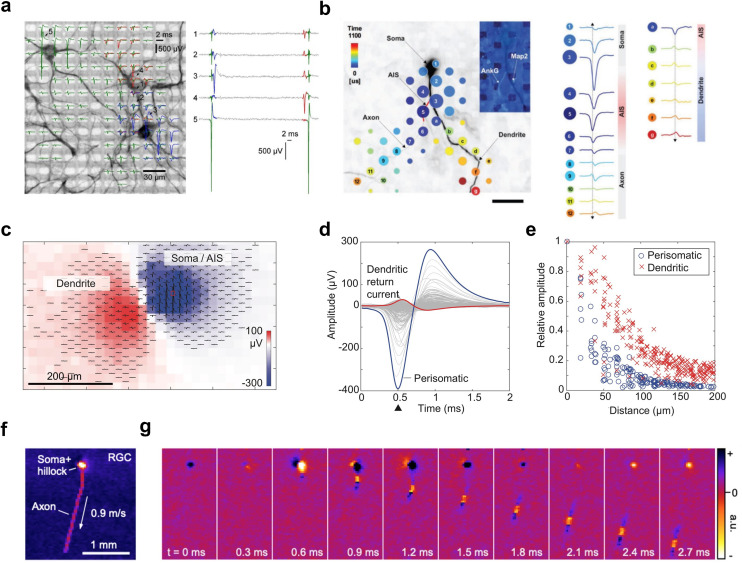
Using high-density microelectrode arrays for extracellular recordings at cellular and subcellular resolution. a, Three primary cortical neurons on a high-density microelectrode array (HD-MEA). The neurons were stained with MAP2 staining (black); the microelectrodes are visible in the background as bright rectangles. The spike-triggered average traces for each neuron are indicated in green, red, and blue. The plot at the right shows example traces of raw data recorded from the three neurons at the indicated locations (see plot on the left). b, The spatial and temporal distribution of the spike-triggered average extracellular action potential (AP) signal of a neuron on an HD-MEA. The neuron has been stained with MAP2 (in black), the axon initial segment (AIS) was stained with AnkG (in red). Dots indicate electrode locations; dot size reflects the peak absolute signal amplitude, and dot color represents the time delay relative to the first voltage peak at the distal AIS. While the area close to the AIS features the largest negative signal amplitude, signals picked up close to the dendrites show positive peaks. Scale bar: 50 μm. c, Example of the extracellular AP signals that can be obtained with today's HD-MEAs. Here, the spatial distribution of the electrical signal of a Purkinje neuron, recorded from an acute cerebellar slice, is depicted. The dendritic part of the signal, comprising mainly positive spikes (in red), and the perisomatic part, comprising predominantly negative spikes (in blue), can be clearly distinguished. d, The same signals as depicted in panel c, but in reference to the time axis (2 ms). e, The relative spike amplitude decay as a function of spatial distance at time point 0.5 ms (see arrow in panel d). Both perisomatic and dendritic signals decay approximately exponentially with distance. Note, the signal was normalized to either the largest positive or negative amplitude. f, An example extracellular signal of a retinal ganglion cell (RGCs) recorded on an APS-based HD-MEA. The recording captured the light-induced spiking activity in the RGC layer of a chicken retina. The panel shows the minimum projection of the signal of a single RGC over approx. 3 ms; the signal travels from the soma/axonal hillock down the axon. Panel g shows the dynamics over time. Images were adapted with permission: panel a was reproduced with permission from ref. 7; copyright (2015): the authors; reproduced under the CC BY 4.0 license. Panel b was reproduced with permission from ref. 24; copyright (2019): the authors, reproduced under the CC BY 4.0 license. Panels c–e were reproduced with permission from ref. 25; copyright (2019): the authors, reproduced under the CC BY 4.0 license. Panels f and g were reproduced (with minor modifications) with permission from ref. 26; copyright (2023): Springer Nature Publishing, reproduced under the CC BY 4.0 license.

**Fig. 2:**
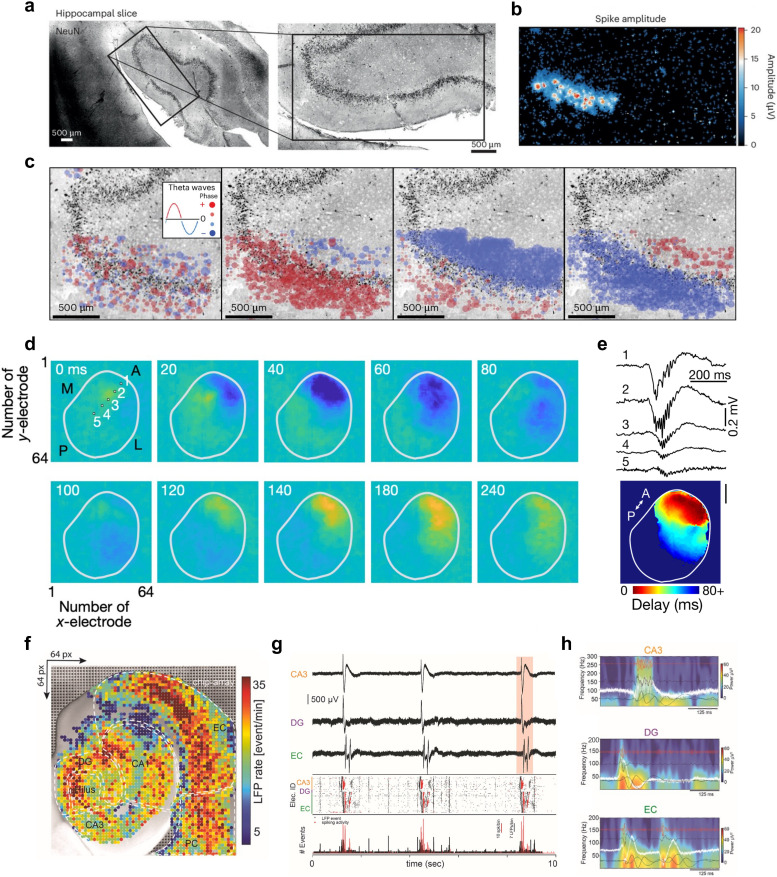
Spatially resolved recordings of neural network dynamics enabled by high-density microelectrode arrays. a, Human hippocampal slice, stained with NeuN (left); the same slice at higher magnification, superimposed with a black rectangle that indicates the size of the recording area of an HD-MEA (right). b, Results of a whole-array HD-MEA activity scan of an organotypic human hippocampal slice; colors indicate the average amplitude values of spikes detected at each microelectrode during the activity scan. The HD-MEA sensing area of the used chip is 3.85 × 2.10 mm^2^. c, During a 0-Mg + kainic acid condition to increase overall activity, human hippocampal slices showed rhythmic bursting activity. Panel c depicts the local field potential dynamics during one of these burst events; red and blue dots indicate the theta wave phase propagation across the HD-MEA; red/blue coloring indicates opposite phases of the theta wave; the size of the dots indicates the magnitude of the phase. d, HD-MEA recording of sharp-wave-ripples (SWRs) in an acute slice of the pallial dorsal ventricular ridge (DVR) of the Australian bearded dragon, *Pagona vitticeps*. Instantaneous voltage images for the SWR event show their initiation near the anterior (A) pole and SWR propagation. Panel e (top plot) shows signal traces of the five small squares in panel d. The lower panel depicts the signal latency with respect to the initiation site. Scale bar: 1 mm. f, LFP event rate of an acute hippocampal-cortical slice on an HD-MEA (sensing area approx. 7 mm^2^). Anatomical regions include the hippocampal subfields (cornu ammonis) CA1 and CA3, the Hilus, the dentate gyrus (DG), the entorhinal cortex (EC), and the perirhinal cortex (PC). g, Three example traces obtained simultaneously from three different regions (CA3, DG and EC) of an acute hippocampal-cortical slice (top panel; recording period 10 s). The traces are aligned to a raster plot (middle panel) that contains both the spike events (in red) and LFP events (in black) during this recording period; an event count histogram of both types of events is provided in the bottom row. Panel h shows pseudo-colored spectrograms for a network event highlighted in panel g (in red); overlaid are band-pass filtered traces for the main oscillatory bands. Panels a–c were reproduced with permission from ref. 27; copyright (2024): Springer Nature Publishing. Panels d and e were reproduced with permission from ref. 28; copyright (2020): Springer Nature Publishing. Panels f–h were reproduced with permission from ref. 29; copyright (2023): Elsevier, reproduced under the CC BY 4.0 license.

**Fig. 3:**
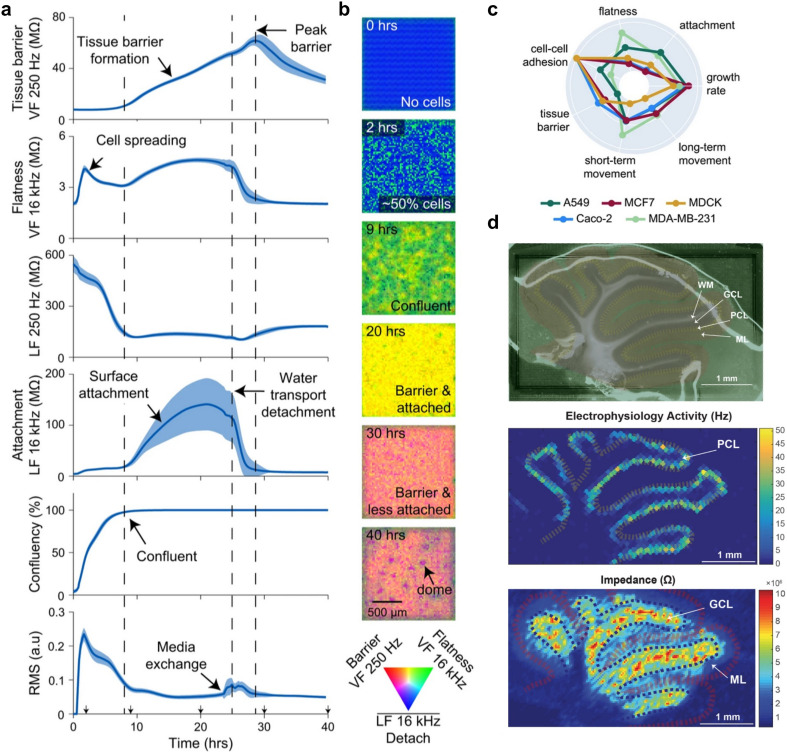
Impedance measurements on CMOS-based microplates and high-density microelectrode arrays. a, Different impedance readouts obtained by imaging Madin–Darby canine kidney (MDCK) cells cultured on CMOS-based microelectrode arrays using distinct electric field configurations. Vertical field (VF) and lateral field (LF) configurations were employed to perform continuous on-chip measurements (every 15 min) of relevant biological parameters (*e.g.*, tissue barrier, cell–cell adhesion, flatness, mobility of cells) from their seeding time point until 40 h post plating. b, An example time course of impedance images of MDCK cells over 40 h (single well). The images were obtained by using the VF 250 Hz (in red), the VF 16 kHz (in green), and the LF 16 kHz (in blue) configurations. c, A radar plot for five different cell lines, based on seven different impedance-based functional parameters, using the approach outlined in panels a and b. d, An acute rodent cerebellar slice attached to an HD-MEA (top plot) for multi-functional imaging; the middle plot shows the spontaneously recorded electrical spike activity (activity scan); the bottom plot shows the corresponding impedance magnitude image of the same slice. Four distinct layers were identified: white matter (WM), containing sparse fibers and axons but no electrogenic cells; the granular cell layer (GCL), densely packed with granule cells; the Purkinje cell layer (PCL), containing highly active Purkinje neurons; and the molecular layer (ML), comprising the flattened dendritic trees of Purkinje cells. Panels a–c were reproduced with permission from ref. 30; copyright (2023): Springer Nature Publishing, reproduced under the CC BY 4.0 license. Panel d was reproduced with permission from ref. 31; copyright (2017): the authors, reproduced under the CC BY 4.0 license.

**Fig. 4:**
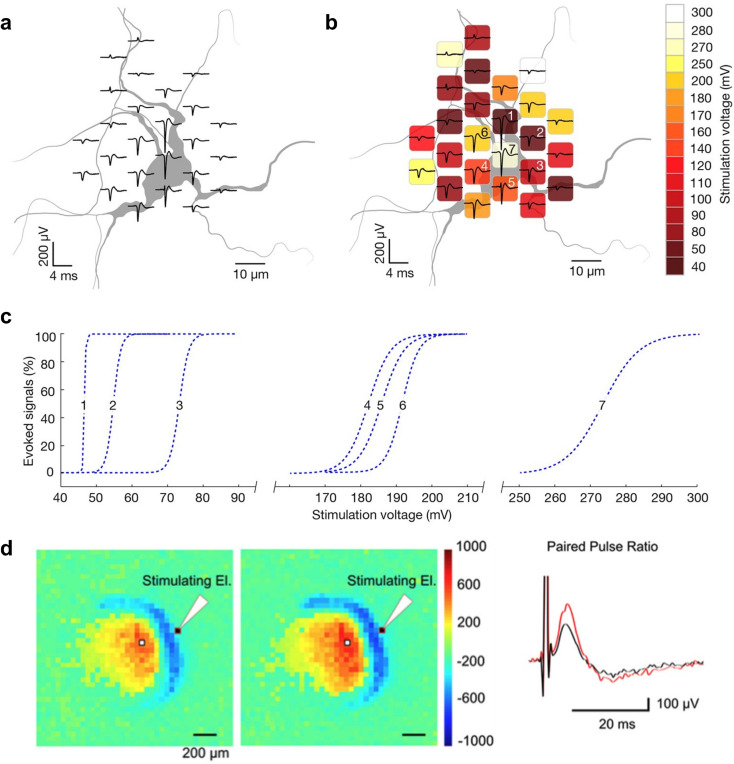
Targeted electrical stimulation of single-cells or circuits on high-density microelectrode arrays to study excitability and plasticity. a, Spike-triggered average extracellular electrical potential footprint of a primary cortical neuron; the footprint is superimposed on a live-cell image of the neuron's morphology, obtained by lipofection. b, Electrode-specific stimulation thresholds for the neuron depicted in a; the colors indicate the activation thresholds. Low activation voltages are found near locations featuring the largest negative signal amplitude within the neuronal extracellular potential footprint. Panel c depicts the excitability profiles and activation thresholds of the seven center electrodes of the stimulation map depicted in panel b. d, The results of stimulating an acute rodent cortico-hippocampal slice on an HD-MEA with an external field electrode. Stimulation was applied to the perforant path of the dentate gyrus (DG), and field excitatory postsynaptic potentials (fEPSPs) were recorded by the HD-MEA. The polarity of the fEPSP corresponded to the anatomical outline of the DG/hilus; the colors indicate dendritic, granule cell and axonal layers (color bar in μV). Electrical images for the first and second pulse after a paired stimulation. The panel on the right shows traces of the first (in black) and second (in red) fEPSP measured after two consecutive stimulations (100 ms apart) – to characterize short-term plasticity in the DG; the electrode from which the measurements were taken is indicated by a white pixel (in both electrical images). Panels a–c were reproduced with permission from ref. 9; copyright (2016): the authors, reproduced under the CC BY 4.0 license. Panel d was reproduced with permission from ref. 32; reproduced under the CC BY 4.0 license.

**Fig. 5:**
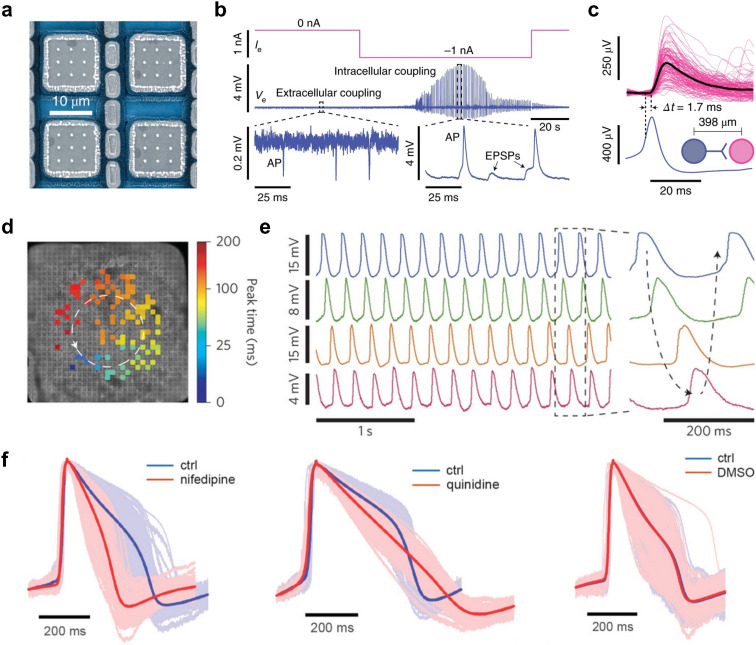
Intracellular-like recordings with 3D-nanostructures on high-density microelectrode arrays. a, Scanning electron microscopy image (top view; false colored) of an electrode structure for intracellular recordings; 3 × 3 Pt-black-coated vertical Pt nanoneedles on a Pt pad electrode. b, Experimental procedure to obtain intracellular-like measurements from primary rodent neurons using Pt-black nanoneedles; the cell was porated by using a pseudo current-clamp circuit (pCC), that allows for injecting a current *I*_e_ (in this example −1 nA). Middle row: following *I*_e_ application, signals transition from extracellular to intracellular-like waveforms featuring action potentials (APs) and excitatory postsynaptic potentials (EPSPs). c, An example putative synaptic connection between a presynaptic (blue) and postsynaptic neuron (magenta), measured by a nanoneedle device. d, An intracellular signal propagation map across cardiomyocytes (CMs) recorded from a CMOS-based nanoelectrode array following an electroporation step. e, Example traces of four different electrodes indicating how the signal propagates over the array. f, The effect of nifedipine (left), quinidine (middle) and the solvent DMSO (right) on the intracellular-like AP waveforms of CMs recorded on HD-MEAs with porous Pt-black microelectrodes. The blue traces indicate the control condition, the red traces show the altered waveform after drug application. Panels a–c were reproduced with permission from ref. 33; copyright (2020): Springer Nature Publishing; panels d and e were reproduced with permission from ref. 34; copyright (2017): Springer Nature Publishing, panel f was reproduced with permission from ref. 35; copyright (2022): American Chemical Society.

This section starts with an overview of the different functionalities of HD-MEAs, detailing (i) how effective voltage, current, and impedance measurements can be realized at high spatiotemporal resolution (Fig. 1–3). Next, we discuss (ii) the stimulation capabilities of HD-MEAs (Fig. 4), and (iii) various electrode connection and read-out schemes. Finally, we report on (iv) recent studies that make use of post-CMOS fabrication and integration techniques to further enhance the capabilities of HD-MEAs (Fig. 5).

### 2.1. Large-scale electrophysiological measurements at high spatiotemporal resolution

MEAs enable functional characterization of cells by monitoring key parameters, such as voltages, currents, impedance, optical properties, and chemical concentrations. Each modality provides distinct insights and contributes to a comprehensive analysis of fundamental physiological and biochemical processes.

#### Voltage measurements

Extracellular voltage recordings are the most commonly used readout mode of HD-MEAs, capturing voltage fluctuations caused by ionic currents flowing across the membranes of electrogenic cells, such as neurons (see Fig. 1 and 2). A key requirement for effective voltage recordings is the minimization of input-referred noise, which is essential for detecting subtle signals at an adequate SNR. Electrode geometry and materials ultimately constrain the SNR, as electrode impedance must be minimized to reduce electrode noise and prevent signal attenuation.^36^ On the circuit side, the input stage of readout circuits must exhibit high input impedance (see below) to avoid signal loss and low noise to maintain signal integrity. To allow for effective voltage/current recordings at high spatiotemporal resolution, HD-MEA designs face several challenges. One major challenge is miniaturization, where circuits must be significantly reduced in size to fit a large number of readout channels within the limited silicon chip area and to enable the integration of electrodes at high density. A second challenge is power efficiency, as the simultaneous operation of numerous channels can generate excessive heat, necessitating careful design to cope with heat dissipation and prevent damage to the biological tissue.

In recent years, various design solutions have been developed to address these challenges.^37–50^ For example, voltage readout circuits often include on-site high-pass filters to remove low-frequency voltage fluctuations, and low-pass filters to eliminate high-frequency components that could cause aliasing during sampling. Moreover, amplification and digitization steps to convert analog signals into digital data have facilitated safe data transfer and storage and efficient post-processing. These steps can be arranged in different ways, with various devices employing different configurations, based on specific application priorities.

Another critical issue with HD-MEA recordings is the substantial volume of generated data. A typical readout channel may sample an electrode voltage at approximately 20 kilosamples per second (kSps) at 10-bit resolution, which produces a data rate of 200 kilobits per second (kbps) per channel. As the number of HD-MEA readout channels can exceed several thousand, the data rate can surpass several gigabits per second, posing significant challenges in data transmission, storage, and post-processing. Recent studies have sought to address this issue. For example, Jang *et al.*^46^ introduced a strategy to reduce data volume using pulse position modulation (PPM)-based active digital pixels (ADPs) combined with a wired-or lossy compression mechanism. Additionally, Cartiglia *et al.*^48^ designed an asynchronous event-based HD-MEA that outputs data only when the electrode voltage changes, which further reduced the data volume.

#### Impedance measurements

Impedance measurements are increasingly recognized as a valuable readout modality of HD-MEAs to assess the composition and properties of biological materials including cells and tissues (Fig. 3).^51–53^ In brief, impedance measurements quantify the complex resistance that a tissue presents to the flow of an alternating current (AC). Impedance is typically measured by applying a small voltage and recording the resulting current – or *vice versa*. Viswam *et al.*^54^ introduced a lock-in-amplifier-based impedance measurement system with 32 channels, which was used to monitor the growth and spreading of embryoid bodies (EBs). The CMOS-based HD-MEA employed in this study also incorporated voltage recording capabilities,^39^ enabling simultaneous recording of cardiac beating. Additionally, the authors performed impedance imaging of acute cerebellar slices, which allowed them to map individual cell layers in the recorded tissue (Fig. 3d, see also ref. 31).

Lopez *et al.*^41^ integrated two distinct impedance measurement strategies in their CMOS-MEA design. The system included 64 impedance spectroscopy channels, capable of analyzing impedance across a wide frequency range (10 Hz to 1 MHz) with high accuracy.^55^ Additionally, a fast impedance monitoring mode was implemented (at 1 or 10 kHz), which enabled rapid detection of impedance variations. Measuring impedance across a wide range of frequencies can provide complementary biological information about tissue and cellular properties. At lower frequencies (approximately 1 Hz to 10 kHz), the measurements reflect how electrical current flows through the extracellular space and around cells, which can provide insights into, *e.g.*, cell contractility and barrier integrity. Conversely, at higher frequencies (above ∼100 kHz), the current can pass through cell membranes, which provides information about membrane properties.

Jung *et al.*^47^ proposed the integration of a four-point impedance measurement technique, which offered advantages over the conventional two-point method. In the two-point configuration, electrode impedance and sample impedance are in series and indistinguishable. The four-point technique uses different electrodes for voltage excitation, voltage sensing, and current sensing, thereby eliminating the effects of electrode impedance and providing a more accurate assessment of the sample impedance.^53^

Chitale *et al.*^30^ introduced a platform for high-content electrical imaging using impedance measurements. Their system integrated CMOS-based HD-MEAs on a 96-well plate, with each well containing 64 × 64 = 4096 electrodes at a pitch of 25 μm (Fig. 3a–c). The system enabled near single-cell resolution measurements and simultaneous recordings from up to 24 wells. By applying various electric field shapes, at different AC frequencies, the authors measured 27 distinct functional and morphological features across several cell types (*e.g.*, cancer cells, epithelial cells) and conducted a compound screen. Some of the extracted features could be linked to biological traits, such as cell motility, confluence, attachment, cell flatness, and tissue barrier integrity – demonstrating the potential of this novel system for high-content phenotypic profiling.

#### Electrochemical detection

Electrochemical detection with HD-MEAs has been used to monitor biochemical analytes, such as neurotransmitters and metabolites in the liquid-phase environment,^56–58^ and previous work has reviewed electrochemical readouts utilizing CMOS technology and monitoring of neurotransmitter release dynamics by means of nano- and microelectrodes.^59–61^ Techniques, such as cyclic voltammetry, can be employed to measure the concentration of molecules by applying a voltage sweep and recording the resulting current, which is indicative of redox reactions occurring at the electrode surface.

For instance, Mulberry *et al.*^44^ presented a system featuring 256 amperometry and fast-scan cyclic voltammetry (FSCV) channels, combined with 256 voltage readouts, facilitating comprehensive electrochemical and electrophysiological measurements. Dragas *et al.*^39^ developed an HD-MEA featuring 28 electrochemical detection units capable of performing FSCV, and reported varying concentrations of dopamine in phosphate-buffered saline (PBS). Finally, Tedjo and Chen^62^ developed a chip featuring 16 064 potentiostat-based electrochemical readouts for chemical imaging. The authors performed a range of microfluidic flow injection experiments (without biological samples) and quantified the sensitivity of their system for detecting neurotransmitter concentrations (*e.g.*, norepinephrine), dissolved oxygen, and pH levels.

#### Other readout methods

In recent years, several novel readout techniques have emerged, offering alternatives to – or enhancements of – traditional methods. These advancements aim to address key limitations, such as sensitivity constraints and scalability challenges, while enabling greater flexibility in experimental applications. While voltage recordings remain dominant due to their simplicity and compatibility with neural and cardiac models, recent advances have demonstrated the potential of current recordings. For example, Abbott *et al.*^63^ reported high-accuracy current recordings using a 64 × 64 electrode CMOS MEA (20 μm electrode pitch), and demonstrated that their method could provide extracellular measurements of small synaptic signals (∼1 pA after averaging) in locations where presynaptic axons and postsynaptic dendrites/somas overlap. Moreover, Lee *et al.*^64^ introduced a multimodal CMOS-based HD-MEA that incorporated dielectrophoresis (DEP)-based analyte enrichment, in which electrical fields were used to manipulate analytes and improve the detection of low-concentration target analytes. The corresponding HD-MEA system included impedance, electrochemical, and optical readouts. Finally, it has been proposed that APs could be detected through the measurement of capacitance changes induced by osmotic shifts.^65,66^

### 2.2. Electrical stimulation

Electrical stimulation is widely used to evoke APs in neuronal or cardiac cells. HD-MEAs, with their small electrodes, enable highly localized and temporally precise stimulation of individual cells – and even allow for stimulation at subcellular resolution, including the targeting of specific compartments within a cell (Fig. 4). This stands in stark contrast to the capabilities of traditional low-resolution MEAs, where fewer electrodes and larger spacing between electrodes result in broader stimulation that typically leads to the activation of larger cell ensembles. HD-MEAs can, for example, be used to stimulate the axon initial segment (AIS), which is particularly advantageous, as the AIS is highly sensitive, and one can trigger APs with minimal stimulation amplitudes.^9,10^ However, there is limited real estate available in HD-MEA devices – that is, the physical space for components in such chip systems is constrained. CMOS designers must therefore balance between integrating many compact, task-specific stimulation units and developing fewer, larger units that support flexible stimulation protocols and incorporate mechanisms to protect both electrodes and cells from potential damage.

#### Voltage- and current-controlled stimulation

Stimulation can be performed using either voltage-controlled or current-controlled methods, each with its own advantages and challenges.^10,67^ The voltage-controlled approach allows for precise control of the electrode voltage, which can help to prevent faradaic processes by keeping the voltage within a safe range.^68,69^ However, the resulting current is dependent on the electrode impedance, which can vary significantly, and the current path is unknown, which may lead to inconsistencies in the tissue volume being stimulated. In contrast, current-controlled stimulation allows direct control over the electrode current, but does not regulate the electrode voltage. This lack of control can result in excessively high electrode voltages that may cause undesirable electrochemical reactions, tissue damage, or electrode degradation. For similar reasons, it is also essential to ensure that the net charge injected into the tissue is zero by applying charge-balancing techniques. This can be achieved by using biphasic current pulses with opposite polarities, along with strategies that guarantee that no net charge remains after stimulation. Systematic studies have been performed to compare voltage and current stimulation strategies, stimulation waveforms, and electrode configurations to optimize stimulation success and to mitigate stimulation artifacts.^10^

Recently, Bertotti *et al.*^38^ introduced a CMOS-MEA with 1024 voltage stimulation channels. In this system, stimulation electrodes were independent and separate from recording electrodes, and stimulation occurred purely capacitively, facilitated by a thin layer of Ti–Zr oxide between the electrodes and the electrolyte. This configuration was reported to reduce the impact of stimulation artifacts. Huys *et al.*^45^ presented a system with 16 384 pixels, each incorporating both voltage recording and dual-mode voltage and current stimulation. The presented device featured sub-micrometer-dimension electrodes shaped like nails, targeted at stimulating individual cardiomyocytes.

#### Closed-loop stimulation

An extension to the traditional voltage- or current-based simulation protocols includes closed-loop systems to interact with neuronal networks. Müller *et al.*^70^ were the first to develop a closed-loop HD-MEA setup, combining the capabilities of HD-MEAs to perform targeted single-cell voltage stimulation at multiple sites (32 stimulation channels) with a field-programmable gate array (FPGA) for sub-millisecond feedback. In this closed-loop system, an FPGA was connected between the host computer and the analog-to-digital converter (ADC) to perform online-spike detection and to instruct when a stimulation should be triggered. In proof-of-concept experiments, the authors then demonstrated that putative synaptic connectivity could be modified by closed-loop feedback stimulation. They reported, that both increases and decreases in correlation-based connectivity strength could be induced.

In a recent study, Wang *et al.*^71^ introduced a CMOS-based integrated circuit that allowed for on-chip closed-loop stimulation with 1024 surface electrodes. Each electrode was connected to one analog front-end unit, supporting both voltage recording and stimulation. The closed-loop stimulation was managed by a global event processor. Moreover, each electrode contained 25 nano-electrodes, arranged in 5 × 5 subsets, which allowed not only for extracellular but also for intracellular-like recordings (*cf.* section 2.4). The authors demonstrated online spike detection and stimulation capabilities in initial experiments with cardiomyocytes and neurons.

In summary, these studies underscore the high degree of versatility, flexibility, and precision of HD-MEAs in electrical stimulation – rendering them a uniquely suited tool for more detailed investigations of electrogenic cells and tissues at both the single-cell and network levels.

### 2.3. Electrode selection

In HD-MEAs, one key design challenge is establishing the connections between the densely packed electrodes and the available readout and stimulation circuits. While the number of circuits is constrained by factors, such as silicon chip area, power consumption, and data volume, the electrode count is limited by array size and electrode pitch. Therefore, as a result, there is often a mismatch between the number of readout or stimulation channels and the total number of electrodes. Here, we briefly outline different strategies that have been developed to address this issue, using either (i) switch matrix (SM) designs, that require the experimenter to select a subset of electrodes for readout and routing of specific functions; (ii) active pixel sensor (APS) designs, that allow readout from all electrodes simultaneously, but at lower SNR; or (iii) dual-mode readout designs, which attempt to combine the first two approaches. A more detailed discussion of these approaches has been provided in a previous review.^14^

#### Selective electrode routing

Electrode routing schemes in HD-MEAs vary significantly in their flexibility. In some systems, readout channels are pre-assigned to specific groups of electrodes, which limits the ability to dynamically select which electrodes can be used. Other designs incorporate a flexible switch matrix (SM), that enables routing readout channels to nearly any electrode, thereby offering great experimental flexibility. Typically, critical circuit components, like low-noise amplifiers and filtering units are placed outside the array, where there are no stringent space constraints, which entails comparably low noise levels (2–3 μV_RMS_). An example of the first approach was demonstrated by Lopez *et al.*,^41^ where the electrode array was divided into 16 wells, each containing a 16 × 16 pixel array, with each pixel comprising a 2 × 2 electrode set. Each pixel included the initial stage of the voltage readout circuitry, along with switches and accessory circuits for stimulation and impedance measurements. Additionally, each pixel featured a 4-to-1 multiplexer to select which of the four electrodes within the pixel was active. Similarly, the readout circuits at high sampling-rate modes reported by Kato *et al.*^72^ and Cha *et al.*^42^ could not be freely assigned to arbitrary sets of electrodes due to the inherent multiplexing structure. In contrast, Frey *et al.*^73^ and subsequent works^7,39,49^ have introduced SM implementations that prioritize flexibility and versatility. In the largest implementation of this matrix,^39^ up to 2048 voltage readout channels, as well as impedance readout and cyclic voltammetry units could be routed to nearly any subset of the 59 760 electrodes, which offered great experimental versatility.

#### Full-frame readout

In full-frame readout architectures, every electrode can be read out simultaneously. One common approach is the active pixel sensor (APS) architecture, where only part of the readout circuit is integrated in the pixel directly beneath the electrode (Fig. 2d and f).^43,45,72^ This design minimizes the distance between the electrode and the input stage, reducing parasitic effects and simplifying the routing process. However, because the available space per pixel in HD-MEAs is limited, the circuits must be highly compact and carefully designed, which often results in higher noise levels (10–20 μV_RMS_). The pixel typically contains only the first amplification stage, with subsequent stages being located at the periphery of the array. Some architectures, such as the systems developed by Jang *et al.*^46^ and Cartiglia *et al.*,^48^ feature readout circuits that perform early digitization or encoding, resulting in a pixel output that is partially digital (*e.g.*, pulse position modulation or event-based encoding). These approaches reduce the complexity of routing analog signals over long distances and enhance overall system performance.

An alternative approach places all readout circuits at the periphery of the array, reserving the area underneath the electrodes solely for routing. The corresponding interconnection scheme can be realized through two strategies: direct routing or fast multiplexing. For direct routing, metallic traces underneath the array can be used to connect each electrode to readout circuits at the periphery. For example, Abbott *et al.*^40^ connected 4096 electrodes directly to peripheral readout circuits using metal lines carefully designed to minimize parasitic effects and signal degradation through attenuation and crosstalk. In contrast, fast multiplexing makes use of switches underneath the array to dynamically connect subsets of electrodes to the peripheral circuits, similar to the SM designs described in the previous section. However, in contrast to the typical SM concept, fast multiplexing allows scanning of the entire array in less than a millisecond. For instance, the system presented by Cha *et al.*^42^ achieved a full-frame sampling rate of 5 kSps. Nevertheless, fast multiplexing introduces challenges, such as noise folding, as the lack of dedicated antialiasing filters for each electrode can degrade the SNR. Additionally, the use of low frame rates may not capture high-frequency signals, which are essential for some biological applications.

Full-frame readout HD-MEAs are advantageous from the experimenter's perspective, as they eliminate the need to select specific electrodes for monitoring and enable comprehensive data collection across the entire array. However, for large arrays, the resulting data volume poses significant challenges for data handling and processing. To mitigate these challenges, some systems reduce the sampling frequency for full-frame readout.^42,49^ While this reduction from the typical 20 kSps to 10 kSps or lower impacts temporal resolution, it can be an acceptable trade-off, depending on the application's need. For example, Kato *et al.*^72^ developed a system with 236 880 electrodes that supports full-frame readout at 10 kSps, generating over 70 Gbps of data. This HD-MEA employs a stacked device structure, integrating three CMOS dies fabricated using different technology nodes: one large die in 90 nm technology and two smaller dies in 65 nm technology. By utilizing a smaller technology node for realizing predominantly digital circuitry, the authors were able to reduce the overall system size. More recently, Suzuki *et al.*^12^ demonstrated the performance of this system through various studies involving brain slices and human induced pluripotent stem cell (iPSC)-derived neuronal cultures.

#### Dual-mode readout schemes

Advanced sampling strategies have been proposed to combine the benefits of full-frame readouts with a SM for low-noise precision recordings. Yuan *et al.*^8,49^ developed a dual-mode system that combined a full-frame readout – offering comparably high noise levels (10 μV_RMS_) and low sampling rate – alongside a smaller set of low-noise channels (2.4 μV_RMS_) that could be flexibly routed to any electrodes *via* a SM. This system enabled high-quality acquisition of signals from the most interesting electrodes, which could then serve as trigger events to guide the recording and analysis of the noisier full-frame dataset. For example, spike-triggered averaging based on precisely timed data from the high-quality channels was used to extract information from the full-frame readout dataset.

### 2.4. Post-CMOS fabrication and integration techniques

Most of the capabilities of HD-MEAs, as described in preceding sections, arise from the integration of dense electrode arrays with advanced CMOS circuitry. The fabrication technologies for CMOS electronics are highly mature, and fabrication is usually done in commercial semiconductor foundries with well-defined design rules and process sequences. On-chip features and structures requiring fabrication steps outside the standardized processes, such as novel nanoscale structures or flexible devices, can only be realized during post-CMOS processing. The use of fully processed CMOS substrates or wafers imposes constraints on post-CMOS processing (*e.g.*, prohibiting high-temperature steps) and often requires unconventional fabrication and integration methods. This section reviews novel HD-MEA functionalities that were realized through such post-CMOS fabrication and integration techniques.

#### 3D nano-electrodes for large-scale intracellular-like neuronal readouts

Traditionally, HD-MEAs have been used to capture extracellular signals of electrogenic cells. However, by modifying planar microscale electrodes into 3D nano-structured electrodes, one can gain intracellular^21^ or intracellular-like^74,75^ (voltage signal amplitudes of 3–15 mV) access to cells (Fig. 5). In neurons, this type of access allows for direct readout of membrane potentials, which enables to resolve not only APs but also much smaller postsynaptic potentials (PSPs, *i.e.*, potential fluctuations below the firing threshold of neurons). Until recently, such subthreshold electrophysiological signals were primarily obtained using sharp microelectrodes or patch-clamp intracellular recordings.^76^ Devices featuring 3D nano-structured electrodes, their modes of intracellular access (*e.g.*, spontaneous penetration, tight engulfment, electroporation or optoporation) and the resulting signals have been reviewed previously.^21,77^

To date, the performance of most intracellular 3D nanoelectrodes has been demonstrated with cardiomyocytes, as their larger size makes facilitates poration. These studies have mostly relied on passive arrays with only a few electrodes, due to the challenges of scaling 3D nanoelectrode fabrication and their integration on CMOS-based chips.^77^ The recent advent of scalable 3D nanoelectrodes, that provide neural intracellular-like readouts, and that can be integrated at large scale on CMOS substrates, represents a major technological advance (Fig. 5). The corresponding HD-MEAs enable massively parallel recordings, but now also provide access to intracellular signaling dynamics, including subthreshold excitatory and inhibitory postsynaptic potentials (EPSPs, IPSPs). Such capabilities enable high-fidelity readouts of the electrical activity of individual neurons and a more thorough investigation of network connectivity. Abbott *et al.*^40^ reported a novel CMOS HD-MEA featuring 4096 monolithically integrated nanostructured electrodes. These electrodes mainly comprised sets of platinum (Pt)-black-coated nanoneedle or microhole designs and – upon applying pseudo-current-clamp or pseudo-voltage-clamp electroporation techniques – allowed for large scale intracellular-like recordings (Fig. 5a–c).^33^ In the best case, 1728 out of 4096 pixel electrodes showed intracellular-like signals, of which 982 could be simultaneously recorded from and used for mapping synaptic connectivity.

More recently, the same group published an optimized CMOS-based 4096 microhole electrode array with significantly improved performance for parallel intracellular recordings.^13^ On average, their devices achieved a 90% intracellular coupling rate (3685 of 4096 pixels), and generally much improved recording performance, such as longer intracellular coupling (>30 min), larger intracellular recordings amplitudes, and the ability to regain access to the same neurons. Using this chip, the authors demonstrated that thousands of putative excitatory and inhibitory connections could be inferred in parallel. If further validated, these microhole HD-MEAs could significantly advance large-scale synaptic connectivity mapping and overcome some of the throughput limitations of traditional intracellular methods.

#### *In vivo* high-density microelectrode probes and flexible substrates

Planar HD-MEAs implemented with silicon-based CMOS technology have predominantly been used *in vitro*, where cells or brain slices are cultured on top of the arrays. In contrast, *in vivo* applications introduce additional requirements that demand specific considerations for the design and fabrication of high-density microelectrode probes. These include, for example, minimizing adverse tissue reactions to these devices by avoiding designs that cause excessive tissue damage during implantation and by preventing chronic inflammatory responses over time.^78^ Moreover, *in vivo* probes must be fabricated from materials that maintain high biocompatibility, mechanical flexibility, and tissue conformability to ensure stable, long-term functional readouts. In addition, the entire probe system needs to be small and lightweight to not interfere with the behavior of the studied animals. Finally, probe architectures should enable recordings from various regions of interest, such as layered or deep-brain structures, as well as across spatially distributed brain areas. Consequently, devices designed for *in vivo* use have taken different technological paths compared to their *in vitro* counterparts. Recent progress, along with an in-depth discussion of design and fabrication considerations for *in vivo* high-density microelectrode devices, has been reviewed elsewhere.^20,79,80^

One approach to leverage CMOS technology for *in vivo* applications involves the development of silicon-based high-density microelectrode probes with monolithically integrated electronics, such as the “Neuropixels”,^81^ “Neuroseeker”^82^ and “SiNAPS”^83,84^ probes. These devices feature high-channel counts in rigid, high-aspect-ratio forms, such as vertical shanks.^85,86^ However, despite their relatively small size and dense electrode packing the rigidity and width of silicon probes – along with movements of anchored probes relative to the brain tissue – can still lead to tissue damage and inflammatory responses during implantation and operation, potentially compromising long-term stability (*cf.* section 5.1). Furthermore, even advanced systems, such as the Neuropixels 2.0 multi-shank probe, featuring four shanks with 1280 recording electrodes per shank (shank pitch: 250 μm, shank length: 10 mm)^3^ can only record from 384 channels simultaneously. Such constraints limit the yield of neurons and the volume from which high-density data can be obtained.^20,87^ Although multiple probes can be implanted in a single animal,^88^ future design improvements are needed to increase the number of readout channels of these devices.

An alternative approach for high-density *in vivo* electrophysiology is the development of microelectrode devices on thin, flexible substrates.^89–91^ These materials conform better to the brain surface and feature less mismatch in mechanical properties between the electrode array and biological tissue. A recent study by Zhao *et al.*^90^ demonstrated that a flexible 3D microelectrode device enabled recordings from up to 1000 neuronal units per mm^3^ of brain volume, with some experiments lasting nearly 10 months. However, soft substrates also introduce new challenges, particularly for scaling up electrode arrays and establishing sufficient connections to supporting circuits. Most current devices still rely on bulky interconnections to circuit boards or application-specific integrated circuits (ASICs), which are not well suited for high-throughput applications.^90,92^ Another approach involves the development of implantable flexible active electronics,^93^ which constitute, however, less mature and less performant technologies than traditional CMOS-based electronics.

#### *In vivo* applications: planar HD-MEAs as backend electronics

Recent developments have also explored approaches that exploit the scalability, versatility, and high performance of CMOS-based *in vitro* HD-MEAs for high-density electrophysiological measurements in living organisms.^87,94,95^ Here, the probes or arrays are connected to and operated by existing CMOS HD-MEAs – now serving as backend electronics – *via* novel connectorization schemes. The interconnect elements are integrated with the implantable electrodes and bonded directly to the backend CMOS-device electrodes, which act as receiving bond pads, thereby enabling robust and scalable connections. The ease and scalability of such connectorization schemes are partly due to the high electrode density and small electrode area of state-of-the-art *in vitro* CMOS HD-MEAs. As long as the pitch of the backend electrodes on the CMOS chip is sufficiently smaller than that of the interconnect elements, precise alignment is not required. These approaches decouple the development of *in vivo* electrode arrays from that of the supporting electronics and enable technological advances that are available in *in vitro* HD-MEAs – such as low-artifact stimulation, improved electrode selection, the availability of impedance measurements or electrochemical detection – to be rapidly adapted for *in vivo* use.

Applying this CMOS HD-MEA backend approach, Kollo *et al.*^87^ and Obaid *et al.*^94^ designed and fabricated bundles of insulated microwires, where the proximal ends were mechanically pressed onto CMOS arrays to form direct interconnections, while the distal ends constituted the implantable, tissue-facing electrode array. Bundles of up to 8640 microwires with 40 μm pitch were connected to CMOS arrays, demonstrating the scalability of the method. Using this scheme, bundles of 200 and 251 wires at ∼100 μm pitch were used to record neural activity from olfactory bulbs of anesthetized mice^87^ and from the motor cortex and dorsal striatum of awake and freely moving mice.^94^ In another implementation, Zhao *et al.*^95^ developed planar flexible polymer devices with up to 2200 electrodes and interconnections to an HD-MEA. The interconnect elements, termed *Flex2Chip*, included microstructures with suspended pads, which adhered to the electrodes of a previously published backend HD-MEA^7^ through capillary and van der Waals forces. This approach enabled an implantable array of 504 electrodes, covering an active area of 760 × 760 μm^2^, to record neural activity from the cortical surface of awake, moving mice. Finally, Wang *et al.*^96^ demonstrated a method for fabricating tissue-penetrating 3D microelectrodes directly onto planar microelectronics by using high-resolution 3D printing *via* 2-photon polymerization and scalable microfabrication. The authors fabricated arrays of 6600 microelectrodes, each with protrusions up to 110 μm in length and 10 μm diameter at 35 μm pitch, and obtained high-fidelity, large-scale retinal recordings with little axonal interference.^96^ Overall, the CMOS HD-MEA backend approach offers a versatile and scalable technique for interfacing silicon microelectronics with *in vivo* neural structures at large scale and cellular resolution.

## 3. Combining HD-MEAs with other experimental techniques

**Fig. 6:**
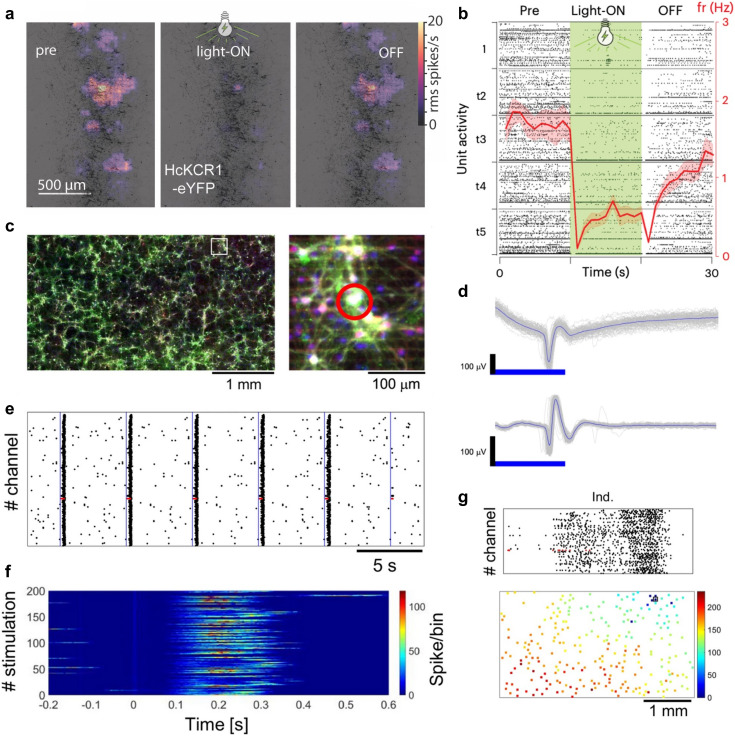
Combining high-density microelectrode array recordings with optogenetic stimulation. a, Heat maps of HD-MEA activity measurements from an organotypic human hippocampal slice, overlaid on a live fluorescence image (neurons express HcKCR1-eYFP, a light-gated potassium channel used for optogenetic silencing). The panel shows how the baseline activity of units (“pre”, left panel) was reduced by optogenetic stimulation (“light-ON”, middle panel), and how it recovered after the light was switched off (“OFF”, right panel). b, Raster plot of unit activity of the slice depicted in a – before, during, and after the optogenetic stimulation (over five trials). The average firing rate of all units during these periods is indicated in red. c, Fluorescence image of a primary rat cortical neuronal network on an HD-MEA (MAP2 in gray, GABA in red, channelrhodopsin-2 (ChR2)-GFP expressing neurons in green, DAPI in blue). The study investigated how optogenetic excitation of single neurons affected network burst activity. d, Direct neuronal responses evoked by repeated optogenetic stimulation of single neurons on the HD-MEA (stimulation area: 50 × 50 μm^2^; stimulation intensity: 15.4 mW mm^−2^). The upper plot shows the signal high-pass filtered above 1 Hz, while the lower plot displays the signal band-pass filtered between 300 and 3500 Hz. Although stimulation induced signal distortions, band-pass filtering mitigated these artifacts. e, Raster plot obtained by optogenetic stimulation of a so-called “leader neuron”. In some primary cortical networks, it is possible to induce network-wide bursts by selective stimulation of leader neurons that may have a hub-role in the network. For example, leader neurons may exhibit many effective connections to other neurons in the network. The blue line indicates the time when the leader neuron was stimulated (the activity of the leader neuron is depicted in red). f, Burst propagation patterns upon stimulation of a leader neuron (200 trials). Time 0 indicates when the leader neuron was stimulated. g, Example raster plot of an optogenetically induced network burst (upper plot). Time is shown on the x-axis, and the channels are plotted along the y-axis; each dot marks a detected spike. In this plot, the network burst was triggered by optogenetic stimulation of a leader neuron, whose activity is depicted in red. The lower plot depicts the spatiotemporal propagation of activity across the whole sensing area of the HD-MEA; colors indicate burst progression from blue (burst onset) to red (burst termination); the position of the leader neuron is indicated by a black square. See also panel c (right) for a fluorescence image of this area on the chip. Panels a and b were reproduced with permission from ref. 27; copyright (2024): Springer Nature Publishing. Panels c–g were reproduced with permission from ref. 97; copyright (2024): Springer Nature Publishing.

**Fig. 7:**
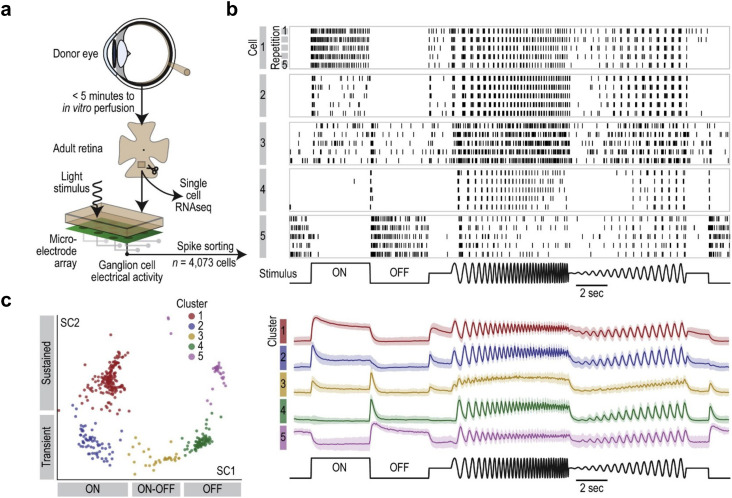
Functional characterization and cell-type classification of human retinal samples on high-density microelectrode arrays. a, Experimental procedures of how adult human retinae can be prepared for functional characterization on HD-MEAs or single-cell RNA sequencing. The tissue was dissected and pressed down on the HD-MEA. HD-MEA recordings from the ganglion cell layer were performed subsequently by applying defined light stimuli to the photoreceptors. Panels b and c illustrate how a series of different stimulus sequences (frequency chirp and intensity sweep, see bottom row) can be used for ganglion cell-type classification. Depicted are the light-induced spike responses of five functionally different cells (each line represents the recorded spikes for one stimulus repetition). c, Clustering map of the functional light responses of different ganglion cells (panel on the left); labels along the *x*/*y* axes indicate the response characteristics of the cells; the average activity of the five inferred clusters is depicted in the panel on the right. Panels a–c were reproduced with permission from ref. 98; copyright (2020): the authors, adopted under the CC-BY 4.0 license.

**Fig. 8:**
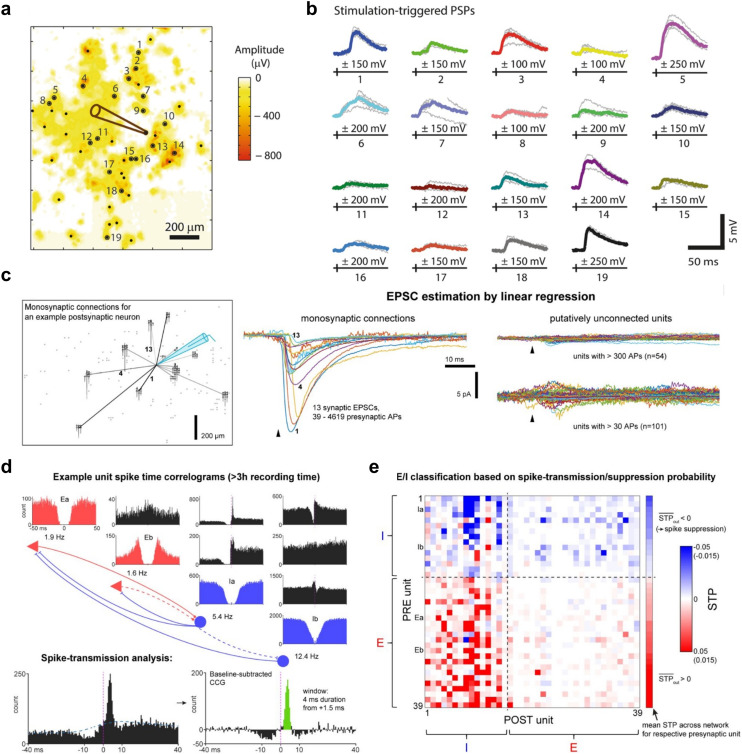
Mapping synaptic connectivity with parallel high-density microelectrode array/patch-clamp recordings. a, Example plot illustrating how targeted electrical stimulation on HD-MEAs can be used to map synaptic connectivity. The panel depicts the result of an HD-MEA activity scan; colors represent the online-detected spike amplitude values for each electrode. Voltage stimulation pulses were then applied to selected sites exhibiting the largest negative signal peaks – corresponding to the putative axon initial segments (AIS) of neurons – and responses were recorded intracellularly with a patch pipette (see pipette drawing). b, The stimulus-triggered postsynaptic potentials (PSPs) for 19 different stimulation sites; individual responses are depicted in gray, average traces in color; PSP stimulation results are depicted for the lowest PSP-evoking stimulation amplitude (in a range from 100 to 250 mV). c, Example connections inferred from a parallel HD-MEA/patch-clamp recording: the plot indicates the patch-pipette on the HD-MEA in blue (schematic), and the electrical footprints (EFs) of 13 putative presynaptic neurons on the array. The middle panel shows the average excitatory postsynaptic currents (EPSCs) of these neurons; the panel at the right shows the signal of unconnected units. Putative connectivity was estimated by a linear regression approach using the spontaneous activity measured through the HD-MEA. d, Auto-correlograms and cross-correlograms (CCGs) of two excitatory and two inhibitory neurons (upper plot), and their putative synaptic connections. The lower plot shows an example CCG of a connected neuronal pair and illustrates how the spike-transmission probability (STP) is inferred from the CCG. e, Example connectivity map showing STP values for a long-term HD-MEA network recording; the labels in this panel (*e.g.*, Ia/b) correspond to those in panel d. Red colors correspond to positive STP values, blue colors indicate negative STP values. Panels a and b were reproduced with permission from ref. 99; copyright (2016): the authors, reproduced under the CC BY 4.0 license. Panels c–e were reproduced with permission from ref. 100; copyright (2024): the authors, reproduced under the CC BY 4.0 license.

In the previous section, we described recent advances in HD-MEA chip design that enabled versatile bidirectional interactions with electrogenic cells, including multimodal readouts across various spatial and temporal scales. While these new HD-MEA capabilities have provided unprecedented means to probe the function of neurons and cardiomyocytes, there are important modalities – for example, optical observation of markers or cellular processes, and mechanical stimulation – that remain impractical to implement directly within CMOS chip designs. To address these shortcomings, HD-MEA technology has been increasingly combined with other experimental techniques. In this section, we first survey different types of HD-MEA combination systems, including integrations with (i) fluorescence microscopy, optogenetics (Fig. 6) and optical stimulation (Fig. 7); (ii) the patch-clamp technique (Fig. 8); and (iii) atomic force microscopy (AFM). We then discuss applications that have exploited the unique features provided by such combined approaches. Finally, we cover studies that integrated HD-MEAs with (iv) microfluidics and surface patterning, *i.e.*, techniques that enable studying biological systems in more controlled microenvironments.

### 3.1. Combining HD-MEAs with optical systems

Simultaneous investigations of neural circuits by means of HD-MEAs and optical microscopy have proven particularly powerful. While both methods can be used to monitor and/or induce neural activity, they are complementary in terms of the information they provide and their respective spatiotemporal resolution. Moreover, quantitative optical readouts of fluorescent reporters can provide estimates of concentrations of important ions (*e.g.*, intracellular Ca^2+^) after careful calibration, which are inaccessible to extracellular electrophysiological recordings.

#### Combining HD-MEAs with fluorescence microscopy

Xue *et al.*^101^ combined HD-MEA network recordings of primary cortical neurons – transduced with genetically-encoded calcium indicators (GECIs) – with confocal microscopy to map the synaptic connectivity of individual neurons. The authors performed confocal imaging of synaptically-evoked calcium transients in small postsynaptic dendritic spines while simultaneously recording network-wide neuronal spiking using HD-MEAs. A correlation-based approach allowed them to link optically measured postsynaptic activity with corresponding presynaptic spiking and, thus, identify individual synaptic connections.

Combining light microscopy with HD-MEAs, more generally, has provided fundamental insights in how neuronal morphology and function are intertwined. Bakkum *et al.*^24^ performed HD-MEA recordings in primary cortical cultures together with fluorescence imaging to probe how the extracellular electrical potential distribution of a neuron – its so-called “electrical footprint” (EF, Fig. 1a) – correlates with known neuronal compartments (Fig. 1b). Interestingly, they found that the proximal axon initial segment (AIS), rather than the soma, produced the largest extracellular AP signal, due to its high ion-channel density. Conversely, immunohistochemical staining after HD-MEA recordings revealed that positive extracellular AP signals often co-localized with dendritic branches (Fig. 1c–e).

In a similar effort, studies have combined HD-MEA recordings with live or *post hoc* imaging to track the initiation, distribution, and reliability of AP propagation along axonal arbors.^102–104^ Such fine-grained alignment of functional readouts with cellular morphology at the subcellular level would not have been feasible with traditional low-density MEAs. Finally, studies also combined microscopy and HD-MEA recordings at the meso-scale, for example, to correlate the layer location of neurons in murine prefrontal cortex slices with their firing properties, excitatory/inhibitory identity, and functional connectivity.^105,106^

#### Optical and optogenetic stimulation on HD-MEAs

Optical probes can be used not only to monitor, but also to control neuronal activity by activating optogenetic actuators, such as channelrhodopsin.^107^ Optogenetics offers an attractive alternative to traditional electrical stimulation, due to its high spatiotemporal precision (*e.g.*, activation or inhibition of individual neurons or circuits at millisecond precision), cell-type specificity (*e.g.*, targeting of specific excitatory or inhibitory neurons), and minimal tissue damage. In combination with large-scale HD-MEA recordings, optogenetic stimulation of selected neurons can help elucidate the contributions of specific neuronal subpopulations to the overall network activity. However, it is important to note that the light intensity levels needed to activate optogenetic constructs are on the order of tens to hundreds of mW cm^−2^, while light stimulation of, *e.g.*, retinal photoreceptors and ganglion cells only requires tens of μW cm^−2^. Consequently, signal artifacts due to changing light intensities may occur – in particular when light impinges on active electronic components like amplifiers. For a more detailed discussion of HD-MEA light sensitivity, see section 5.2.

While optogenetics has been combined with traditional MEAs to study closed-loop neural control,^108^ short- and long-term plasticity,^109,110^ and encoding of stimulus-specific information,^111^ – its application to HD-MEAs is still relatively nascent. Nevertheless, proof-of-principle studies have demonstrated it's potential. For example, in retina explants, retinal ganglion cell (RGC) spiking was recorded on HD-MEAs while being simultaneously modulated by optogenetic stimulation.^112,113^ Optogenetic stimulation on HD-MEAs has also been used as ground truth for the refinement of spike-sorting algorithms.^114^ Andrews *et al.*^27^ recently performed HD-MEA recordings in human hippocampal slices obtained from patients with drug-resistant temporal lobe epilepsy (Fig. 2a–c), and studied how AAV-mediated optogenetic stimulation could modulate spontaneous and drug-induced electrical activity (Fig. 6a and b). Finally, recent work by Kobayashi *et al.*^97^ introduced an optogenetic stimulation setup that employs a digital mirror device (DMD) to selectively stimulate individual neurons on HD-MEAs (Fig. 6c–g). The authors systematically evaluated the artifacts associated with optical stimulation on the HD-MEA, including the effect of stimulation intensity, duration, and the area of illumination. Their results underscore the high spatial and temporal resolution achievable with DMD-based optogenetic stimulation (*e.g.*, single-neuron stimulation was achieved by a 50 × 50 μm^2^ illumination field, and 5 ms light pulses). Furthermore, they demonstrated how targeted optical stimulation can be leveraged to study the role of specific neurons (“leader neurons”) in shaping network activity (Fig. 6e–g).

Another important experimental setup entails light-induced stimulation of retina explants mounted on HD-MEAs for *ex vivo* studies of retinal function. HD-MEAs have been applied to study retinal information processing and functional characterization of RGCs, across a range of species, including mouse,^115–117^ rabbit,^118^ chicken,^26^ pig,^119^ macaque,^120^ and even human retinae (Fig. 7).^98^ Here, light stimuli of comparably low intensity, which did not produce signal artifacts, were projected onto the retina explant to directly activate photoreceptors, while recording their spiking simultaneously on the HD-MEA.

To obtain light-induced stimulation profiles of specific cell types, studies used different repeating visual stimulation patterns that enabled the inference of receptive field properties, direction selectivity and variations in AP propagation velocities (Fig. 1f and g and 7).^113^ Acute retinal recordings have also been used for disease modelling, *e.g.*, to probe functional alterations in RGC direction selectivity in congenital nystagmus.^117^ While low-density MEAs have historically been used and continue to be used in retinal studies, modern HD-MEA systems offer significant advantages in terms of electrode density, routing flexibility and spike-sorting performance. The data obtained from HD-MEAs enable high-resolution investigations at cellular and subcellular level, providing deeper insights into the functional dynamics of retinal cells and their complex interplay. As a result, HD-MEAs are increasingly becoming the preferred measurement system for *ex vivo* retinal studies, as they offer a more detailed and comprehensive picture of retinal function and pathology.

### 3.2. Combining HD-MEAs with patch-clamp recordings

While HD-MEAs enable the study of neuronal network activity at single-cell or even subcellular resolution, they do not provide a complete view of the full repertoire of electrophysiological signals. Specifically, small subthreshold signals – such as excitatory and inhibitory postsynaptic potentials (EPSPs, IPSPs) – are usually not detectable (though see section 2.4 for recent advances in CMOS-based microhole/nanoneedle electrode arrays that provide intracellular-like recordings). Moreover, while HD-MEAs can be used to measure differential voltage variations, they do not offer access to absolute membrane potential values, which are only attainable through intracellular recording techniques. Therefore, studies have combined HD-MEAs with whole-cell patch clamp (Fig. 8), a technique that is still regarded as the gold standard for intracellular electrophysiology.

Jäckel *et al.*^99^ introduced a methodology for parallel HD-MEA/patch-clamp recordings and demonstrated the power of this combined approach by investigating synaptic connectivity and plasticity in primary neuron cultures (Fig. 8a and b). They stimulated neurons extracellularly *via* HD-MEA electrodes and recorded evoked postsynaptic responses with a patch-clamp pipette. This confirmed monosynaptic connections and allowed estimating synaptic strength. Furthermore, to study short- and long-term synaptic plasticity, the authors applied paired-pulse protocols and intracellular tetanization. By patching a neuron on the HD-MEA and then applying electrical stimuli sequentially to putative presynaptic neurons (*i.e.*, neurons presumed to evoke postsynaptic potentials in the patched neuron), they identified several neuron pairs that exhibited short-term facilitation.

Building on this setup for parallel HD-MEA/patch-clamp measurements, Bartram *et al.*^100^ developed a linear regression approach to map putative monosynaptic connections to individual patched neurons using large-scale recordings of spontaneous network activity (Fig. 8c). In addition, the study used multi-hour network recordings to infer spike-transmission probabilities (STPs) between neurons (Fig. 8d), to classify neurons into excitatory and inhibitory types (Fig. 8e), and to uncover synaptic events that controlled postsynaptic spike timing. In other studies combining HD-MEA and patch-clamp recordings, intracellularly recorded APs served as “ground truth” to verify the timing and waveform characteristics of extracellularly detected APs: For example, such data was instrumental in validating spike-sorting algorithms,^121^ assessing the spatial spread of extracellular APs,^122^ and constructing improved neuronal multi-compartment models based on HD-MEA data.^123^ Together, these studies demonstrate that many experimental questions benefit from an integration of HD-MEAs with intracellular recording techniques to investigate aspects of cellular and network function that HD-MEAs alone cannot currently address.

Beyond validating extracellular recordings, patch-clamp recordings offer access to key biophysical parameters, including absolute membrane potentials, the activation and inactivation dynamics of specific ion channels, the reversal potentials of individual ion species, and even single-channel activity (in cell-attached mode). Moreover, patch clamp enables targeted manipulation of cells by introducing compounds through the patch pipette, allowing controlled mechanistic studies of how drugs affect specific intracellular processes. Such insights can only be indirectly obtained through HD-MEA recordings. Thus, parallel HD-MEA/patch-clamp measurements offer critical information on fundamental properties of neuronal signaling across multiple scales, enabling a more comprehensive and mechanistic understanding of how the state and excitability of individual cells correlates with observed network-level behavior.

### 3.3. Mechanical stimulation of cells on HD-MEAs

A growing body of evidence shows that neurons may be mechanosensitive cells,^124^ which has motivated the development of platforms that combine HD-MEAs with tools for delivering mechanical stimuli. HD-MEAs enable precise measurements of mechanically induced neuronal spiking without introducing additional mechanical stress as might occur, for example, with the use of patch-clamp techniques.

Using such a combined system, Marrese *et al.*^125^ applied micro-indentations to mechanically stimulate the photoreceptor layer of retina explants and observed a modulation of RGC activity, that could be detected by simultaneous HD-MEA recordings. To achieve subcellular resolution for both stimulation and electrical readout, Kasuba *et al.*^126^ recently combined an AFM with an HD-MEA platform to investigate the electrophysiological responses of individual neurons to mechanical stimulation. The authors found a differential mechanosensation of neurons depending on the precise stimulus characteristics: transient mechanical stimulation at the soma was found to evoke APs and modulate the mean neuronal firing rate following multiple transient compressions, whereas neurons displayed a broad resilience to static compression.

Techniques for the precise physical targeting of cells provide valuable complementary insights when combined with HD-MEAs. Moreover, positioning HD-MEA devices beneath the neuronal preparation preserves access to the tissue for mechanical manipulation. Nevertheless, the stiffness and rigidity of HD-MEAs (typically in the GPa range) may affect the outcome of mechanical and electrophysiological measurements as, *e.g.*, developing neurons and cardiomyocytes are used to significantly softer environments (*cf.* section 5.1).

### 3.4. Combining HD-MEAs with microstructures, surface patterning, and microfluidic systems

A wide range of microfabrication techniques has been developed to improve the physiological relevance of *in vitro* neural circuits (see ref. 127 and 128 for recent reviews on these techniques, common microfluidic devices, and their combination with traditional MEAs). Strategies for generating more organized or oriented neural networks include, for example, structural confinement (*e.g.*, by compartments, microchannels or microtunnels)^129,130^ and chemical surface patterning (*e.g.*, by microcontact printing and UV photolithography).^131,132^ A primary goal of these approaches is to define areas of neuron adhesion and to spatially confine neuronal growth after plating, as well as to compartmentalize networks into “nodes” for further study.

Physical confinement through polydimethylsiloxane (PDMS) or hydrogel microstructures is widely regarded as a reliable technique to engineer *in vitro* neural networks,^133^ and many studies have used traditional MEAs to investigate the activity and connectivity of such compartmentalized neural networks.^130,132,134–137^ A popular design that has been used with standard MEAs is a two-compartment system.^138^ It enables to spatially separate neuronal somas and axons and facilitates the co-culturing of different neuronal subtypes so that cell-type-specific connectivity and dynamics can be investigated.^139–142^ Several studies expanded on this idea and developed protocols to probe multi-node networks.^132,134–136,143–144^ While microstructures allow control over the seeding location of cells, the gross architecture of engineered networks, and local drug stimulation^146^ – microchannels provide additional means to guide the directional growth of axons, and hence axonal signal propagation in a network.^132,135,147,148^ Varying the number of these microchannels enables to manipulate the connection strength between neural populations.^144^ Microchannels have also been combined with microgrooves to define tissue morphology and to precisely control the location of cells on MEAs.^149^

Some of these patterning approaches have now been translated to HD-MEAs. Using HD-MEAs in conjunction with microstructures bears several advantages: it offers near-complete coverage of the dynamics of small, confined networks; it enables high spatiotemporal resolution readouts of how spatial constraints affect electrical activity; and it facilitates studying how specific inputs to the network, such as the stimulation of selected neurons, lead to distinct network-response patterns. A pioneering first study by Lewandowska *et al.*^150^ integrated a CMOS-based HD-MEA (11 011 electrodes, electrode size 6 × 8 μm^2^, 17 μm center-to-center pitch) with a two-chamber PDMS structure connected by microchannels. They demonstrated precise tracking of axonal APs over several days of development and provided a detailed view of how axonal propagation dynamics and axonal AP shapes are modulated by spatial confinements.

More recently, Duru *et al.*^133^ developed techniques to improve the adhesion of microfluidic devices on HD-MEAs, enabling the integration of more sophisticated PDMS microstructures with commercial HD-MEAs to create circular 4-node networks. Later, the same group developed even more refined microfluidic structures and applied targeted stimulations to map out parameters that determine stimulation-induced electrical activity in neuronal networks obtained from rodent primary cortical cultures.^151^ They also explored retinal spheroids in a bio-hybrid microfluidic axon guidance system,^152^ and probed the axonal electrophysiology of human iPSC-derived sensory neurons.^153^

While these studies focused on combinations of HD-MEAs with static microstructures, there has been little work on integration HD-MEAs with perfused microfluidic chips. Bounik *et al.*^154^ recently reported on a multifunctional CMOS-based HD-MEA system featuring two arrays of 1024 electrodes (electrode area: 38 × 42 μm^2^; 1.6 × 1.6 mm^2^ sensing area per array) that was integrated into an open microfluidic chip. The HD-MEA included functional units for impedance spectroscopy, electrophysiological measurements, electrochemical sensing, and electrical stimulation. The setup could be operated in two different experimental modes: a hanging-drop mode (with the electrode array positioned on the ceiling substrate) and a standing-drop mode (with the electrode array on the bottom). The authors then performed proof-of-concept measurements from 3D microtissues composed of human iPSC-derived cardiomyocytes. While promising, the assembly and maintenance of such hybrid devices can be challenging, and it is important to consider the potential effects of a dynamic microfluidic environment (*e.g.*, the shear stress on cells induced by variations in fluid flow, or the impact of evaporation on tissue positioning) on electrophysiological readouts.

## 4. Approaches to analyze HD-MEA data

Recent advances in HD-MEA technology, along with the integration of different recording modalities, have resulted in the generation of large and complex datasets. As outlined in the previous chapters, the high spatiotemporal resolution of HD-MEA recordings offers unique opportunities to investigate functional properties of electrogenic cells/tissues across multiple scales, ranging from subcellular compartments and individual cells to entire networks. In this chapter, we (i) describe key signal components observed in extracellular HD-MEA recordings of neuronal and cardiac cells, and (ii) present well-established data-processing strategies that enable researchers to navigate this complex data landscape and extract biologically relevant insights. Finally, (iii) we discuss studies that have combined HD-MEAs with monolayer and organoid-derived neuronal networks to probe learning behaviors *in vitro*. While HD-MEAs have also been used for a range of impedance-related measurements (*cf.* section 2.1; Fig. 3), this topic has been reviewed elsewhere.^53^

### 4.1. Signal content

The extracellular signals recorded from neuronal networks using HD-MEA consist primarily of two components: local field potentials (LFPs) and action potentials (APs or spikes), both reflecting neuronal activity in the vicinity of the electrode. LFPs, typically extracted by filtering signals below ∼300 Hz, represent the combined synaptic and subthreshold activity of neurons within a few hundred micrometers of the electrode. These lower-frequency signals capture slower events, such as synaptic integration, and are detectable over a larger radius due to reduced attenuation by the extracellular environment. LFP amplitudes range from a few microvolts (μV) to millivolts (mV) and are typically smaller *in vitro* than *in vivo*. Although the relationship between subthreshold synaptic potentials (*e.g.*, EPSPs and IPSPs) and LFP dynamics has been investigated using combined intra- and extracellular recordings, extracting these small signals from extracellular HD-MEA recordings alone remains cumbersome.^63^ Extracellular electrophysiological signals between ∼300 Hz and 6000 Hz are referred to as multi-unit activity (MUA) and primarily reflect APs from neurons located within tens of micrometers from the electrode. Due to their rapid spatial decay of APs (Fig. 1b–e), APs are detected only from nearby cells, which is critical for de-mixing the activity of individual neurons (see below). The duration of neuronal APs is 1–2 ms, and their signal amplitudes are in the range of tens to hundreds of μV.

In addition to neuronal signals, HD-MEAs are increasingly used to measure the bioelectrical activity of other electrogenic cells. The signals of cardiac cells, for example, originate from an interconnected cardiac syncytium and differ markedly from neuronal signals. The duration of cardiac potentials is longer (∼200–400 ms), and the signal amplitudes of cardiac cells are higher (in the mV range). Moreover, cardiac signals exhibit a lower and broader frequency spectrum, typically analyzed in the range of 0.1 Hz to 1 kHz. Similarly, HD-MEA recordings from non-neuronal tissues – such as muscle and pancreas - show distinct amplitude profiles, time scales, and spectral characteristics (see, *e.g.*, ref. 155).

#### Local field potentials

Local field potentials are thought to reflect the input to local neuronal networks.^156,157^ They capture subthreshold and network-level processes, and different frequency bands have been linked to specific functions during motor control, attention, and sensory integration *in vivo*.^158^ Their stability in chronic recordings makes LFPs particularly suitable for long-term monitoring and neuroprosthetic applications.^159^ However, due to their complex, multi-source origins and ambiguous relationship to APs, interpreting LFPs remains a matter of ongoing debate.^160^

HD-MEAs, thanks to their large sensing areas and high electrode densities, enable a very detailed analysis of the spatiotemporal properties of LFP signals (see Fig. 2). LFP recordings using HD-MEAs (and other large-scale MEAs) have been performed across a range of different species (*e.g.*, mouse, rat, and human) and in different *ex vivo* slice preparations, including the hippocampus (Fig. 2a–c),^12,29^ cortico-hippocampal slices (Fig. 2f–h),^12,161^ somatosensory cortex,^161^ claustrum (Fig. 2d),^28^ and the olfactory bulb.^162^ Recently introduced HD-MEAs, such as the chip by Suzuki *et al.*^12^ with a 5.5 × 5.9 mm^2^ active area, even allow for simultaneous measurements across multiple regions, spanning all layers of the cerebral cortex along with midbrain, caudate, and thalamic structures.

HD-MEA measurements from slice preparations preserve key morphological and electrophysiological features of *in vivo* tissue. Additionally, these recordings can be aligned with microscopy images of the respective biological preparations (Fig. 2), enabling detailed spatial correlations. For example, HD-MEA recordings facilitated the study of spontaneous sharp-wave ripple (SWR) propagation in acute *ex vivo* slices of the reptilian claustrum (Fig. 2d).^28^ In this study, the authors demonstrated that SWRs propagated from the anterior medial to posterior lateral poles of the dorsal ventricular ridge (DVR), in a manner similar to what has been observed *in vivo*.

LFP analyses probing different frequency bands, such as theta band oscillations, have also been performed in other *in vitro* systems, including human organotypic hippocampal slices and cerebral organoids (*cf.* Box 2).^5,163^ It should be noted, however, that LFP signals observed in such *in vitro* preparations may differ from the oscillations found in the living animal – both in their underlying mechanisms and patterns – as key brain regions involved in generating *in vivo* oscillatory dynamics are lacking in these preparations. Nevertheless, optogenetic stimulation has been successfully used to induce theta-nested gamma oscillations in entorhinal–hippocampal circuits of acute rodent slices on traditional MEAs,^164,165^ and HD-MEA recordings from organotypic human hippocampal slices have shown increases in theta frequency during pharmacologically induced network activity (Fig. 2).^27^ The capabilities of today's HD-MEAs offer great potential for advanced LFP analyses and will likely contribute to a deeper understanding of the mechanistic underpinnings of these complex signals.^166^

**Box 2: HD-MEA recordings from brain organoids**

**Brain organoids** are self-organizing, three-dimensional (3D) structures derived from human induced pluripotent stem cells (iPSCs) or embryonic stem cells (ESCs) that recapitulate aspects of the cellular diversity and cytoarchitecture of the developing human brain. Reductionist in nature, organoids bridge a critical gap between traditional monolayer cell cultures and animal model, by offering a human genetic background and increased tissue complexity. Recent refinements in organoid protocols have enabled the development of region-specific models, including those with predominant midbrain,^167^ thalamic,^168^ cerebellar^169^ and retinal^98^ identities. There is hope that these models will deepen our understanding of human brain development and improve disease modeling.^170^ Patterned organoids have been fused into so-called “*assembloids*”, such as cortico–thalamic^171^ or midbrain–striatal–cortical organoids,^172^ further enhancing model complexity and expanding potential applications. Because electrophysiological assessment of brain organoids provides insights into neuronal connectivity and network dynamics, a range of techniques – including calcium imaging, patch-clamp, and traditional MEA recordings – have been applied to study their functional properties (see ref. 173 for a review). However, each method comes with its own advantages and limitations.

An increasing number of studies have applied **HD-MEAs** to electrophysiologically probe brain organoids and to record and stimulate them at high spatiotemporal resolution (Fig. 10). Schröter *et al.*^5^ first reported HD-MEA recordings of sliced human cerebral organoids. Their study demonstrated that 3–4-months-old organoid slices showed intrinsic electrical activity and that individual neurons could be tracked over multiple days on the chip. They also found that the AP propagation velocity in human organoids was comparable to those previously observed in human neurons in monolayer culture^6^ and that network activity could be modulated by drugs acting on GABA_A_ and NMDA/AMPA receptors.^5^ Sharf *et al.*^163^ studied neuronal firing, functional connectivity, and theta-like oscillations in human brain organoids, and demonstrated their modulation by benzodiazepines. Moreover, numerous HD-MEA studies have explored the functional phenotypes of human brain organoids, obtained from human iPSCs of individuals with neurological disorders, such as Rett and Dravet syndrome.^174,186^ Finally, as reviewed in section 4.3, there is also growing interest in combining HD-MEAs with organoids to study bio-computing.

Despite their potential, planar HD-MEAs also have notable limitations when employed for organoid electrophysiology. For example, recordings primarily capture the activity at the outer surface of organoids, leaving critical inner regions of the tissue inaccessible. Moreover, the long maturation time of human brain organoids, coupled with the need for stable attachment of the tissue to the array, poses challenges for tissue maintenance (*e.g.*, lack of perfusion at the chip-tissue interface, flattening of organoids, and increased stress during culturing). To better address some of these limitations, advanced MEA technologies and refined culturing strategies have been introduced (see ref. 175 for a recent review). For example, 3D HD-MEAs have been used for recordings from deeper tissue layers,^176,177^ and different kinds of flexible and stretchable MEA devices have been applied for chronic recordings from organoids.^178–180^ In addition, new organoid culturing techniques – such as the air–liquid-interface (ALI) culture of sliced organoids – have been developed to improve the functional characterization of organoids,^181^ and automated culturing platforms have been introduced to enable long-term organoid maintenance and HD-MEA recordings.^182^

#### Spike activity

There is a large body of studies that has successfully used standard, low-density MEAs to functionally characterize the spike activity of electrogenic cells.^130^ However, the large electrode size and wide spacing of these devices make the inference of single-cell activity difficult. As a result, many studies applied simple threshold criteria to define AP spiking, relying on MUA as a proxy for neuronal activity. If the primary goal of a study is to assess overall activity patterns or to analyze population-level dynamics, MUA can provide a robust measure – in particular when the activity of neuronal ensembles is low-dimensional.^183^ MUA-based firing statistics have been widely used to describe neuronal network development,^6,184^ differentiate between cell lines and cell types,^6,185^ and study human cellular models of neurological diseases.^6,186–188^ However, because the MUA signal represents a superposition of the activity of multiple neighboring neurons, its interpretability is somewhat limited.

**Fig. 9:**
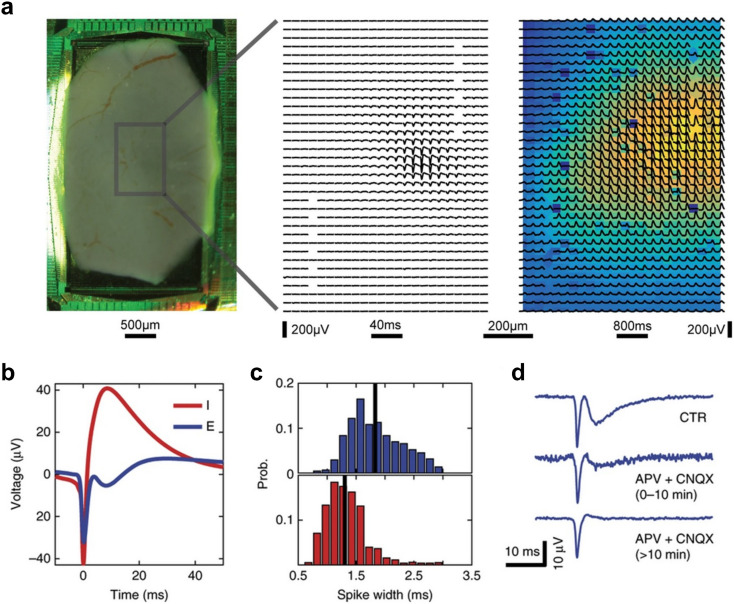
Cell-type classification on high-density microelectrode arrays. a, A turtle cortical slab placed on top of an HD-MEA (left). The middle panel provides a zoomed-in view of a subset of recording electrodes of an HD-MEA, as indicated by the black rectangle in the left panel. It displays a high-pass filtered (>200 Hz) spike-triggered extracellular template, generated by averaging 500 spikes from a single unit; each channel trace represents a 10 ms segment. The right panel shows the spike-triggered average of voltage traces with open filters (1–3500 Hz) from the same unit. Here, each channel depicts 200 ms of signal, starting 100 ms before the detected spike. The authors referred to the slower signal that follows APs as a spike-induced field (SIF). These SIF waveform traces can be used for cell-type classification. Average SIF waveforms of excitatory (E) and inhibitory (I) units demonstrate clear separability between both cell classes based on SIF features. c, Histograms of spike widths of putative E/I neurons, classified by their SIF polarity; colors as in panel b. d, The negative SIF waveform of a pyramidal neuron on an MEA can be modulated by AMPA and NMDA receptor antagonists (CNQX and APV). Of note, the SIF waveforms shown here were evoked by current injection through a patch-clamp electrode. Panels a–d were reproduced with permission from ref. 122; copyright (2017): Springer Nature Publishing.

In contrast, data recorded with HD-MEAs can be further processed using spike sorting to decompose MUA into the contributions of individual neurons (*cf.* section 4.2). The resulting single-unit activity provides the spike timing and the identities of neurons located in the neighborhood of the recording electrodes. In practice, the choice between analyzing single-unit or multi-unit activity often involves a trade-off between computational complexity and the level of detail required. Many advanced computational routines, such as connectivity inference (Fig. 8), reconstruction of axonal AP dynamics (Fig. 1b and f), and cell-type classification (Fig. 7 and 9), rely on spike-sorted single-unit data.

### 4.2. Computational routines for HD-MEA data analysis

#### Spike sorting

After filtering the raw electrophysiological signals, spikes can be detected in HD-MEA recordings using a variety of methods, including amplitude thresholding, template matching, and wavelet-based approaches.^189–191^ Spike sorting is then the analytical step to disentangle the contributions of individual neurons from the mixed extracellular signal, enabling a comprehensive and spatially resolved functional characterization of neuronal networks. The typical spike-sorting workflow begins with band-pass-filtered extracellular signals that have been preprocessed to extract MUA. Because densely packed micro-electrodes tend to record correlated noise, whitening procedures are applied to de-correlate the signals. Spike waveforms are then detected through threshold crossings, followed by temporal alignment and projection into a lower-dimensional space, such as the principal component (PC) space. Next, clustering algorithms are applied to segregate spikes into clusters, each corresponding to a putative individual neuron. From each cluster, a spatially distributed spike template (*i.e.*, the average extracellular AP waveform of a unit) can be derived. Iterative refinement of the data using template matching algorithms can further enhance the accuracy of single-unit spike identification.^191–193^ The high spatiotemporal resolution of modern HD-MEAs increases the yield and precision of spike sorting, enabling robust results even for neurons that are in close proximity. However, the dense packing of electrodes and the large data volumes recorded with HD-MEAs also present significant challenges for most currently available spike-sorting algorithms.^193^

#### Quality control and data curation

Although spike-sorting procedures have been employed in neuroscience for several decades,^194^ and numerous algorithms have been published,^195–198^ no consensus has emerged on the best implementation, and various workflows with different underlying assumptions are in use (for reviews, see ref. 193 and 199). As a blind source separation problem,^191^ the validation of spike-sorting algorithms poses a significant challenge and is, in the absence of realistic ground-truth data, mostly performed on either single-cell HD-MEA/patch-clamp recordings or synthetic data.^197,200–202^ However, even for such benchmark datasets, results obtained with different spike-sorting algorithms differ widely, and the performance often depends on specific characteristics of the recording, such as the degree of network synchronization (which can lead to overlapping spikes and reduced sorting accuracy).^200,203^

To address these challenges, post-processing techniques are commonly applied, including automated quality control workflows that identify high-quality units based on biologically relevant parameters – such as AP shape (*e.g.*, maximum duration), firing patterns (*e.g.*, activity within the refractory period), and separability from other units (*e.g.*, in PC space).^200,204,205^ Related approaches have recently been introduced to identify and match units recorded at different time points, facilitating the tracking of network and single-cell activity across development.^206^ Still, if the data originate from a heterogeneous neuronal population, selecting appropriate parameter values is challenging and may result in a bias towards certain neuron types. Therefore, while not feasible for large datasets, manual curation through interactive data visualization tools, such as the publicly available software suite *Phy*,^207^ remains a commonly used post-processing step. Once high-quality single-unit spike trains have been isolated, a range of techniques can be applied to quantify firing statistics, infer connectivity, and characterize neuronal network dynamics.

#### Analysis of network bursts, waves, and avalanches

Spontaneous correlated firing patterns, commonly referred to as network bursts (NBs), are an important developmental hallmark of neuronal systems and have been observed across many species.^208^ Early spontaneous activity patterns play important roles in fundamental processes during mammalian brain development, including the refinement of axonal projections,^209^ the specification of neurotransmitters,^210^ and programmed cell death.^211^ As such, they provide important insights into the developmental and functional state of neuronal networks.^212^ Despite their prominent role and widespread use in characterizing neuronal activity, there is no standard definition of what constitutes a NB. Furthermore, no burst detection method so far generalizes well across different experimental settings, and most algorithms require manual parameter tuning (*cf.* Box 3).^213^

**Box 3: Algorithms for automated network burst detection**

Despite their widespread use in characterizing neuronal activity *in vitro*, there is no universally accepted definition of bursts, and the criteria used to detect them vary across experimental contexts. As a result, numerous burst-detection methods have been developed over the years, many of which perform burst detection on individual channels, which then have to be aggregated into network bursts (NBs) in a separate step. Most approaches fall into two categories: **threshold-based** and **surprise-based** methods.^213,214^

**Threshold-based methods** typically involve imposing a threshold on activity metrics, such as the firing rate or the maximum interspike interval (ISI), with the threshold value often determined by visual inspection.^215,216^ Additional parameters – such as the minimum burst duration – have been utilized to restrict NB detection to biologically meaningful events.^217^ Other methods adaptively infer thresholding parameters, *e.g.*, based on features of the ISI distribution (see ref. 218 and 219). The first two peaks in the distribution ideally correspond to ISIs within and outside of bursts, with the trough in between serving as a potential event detection threshold. Considering every *n*th network-wide spike for the ISI calculation was later shown to improve detection performance.^218^

**Surprise-based methods**, on the other hand, detect bursts by quantifying deviations from an assumed firing-rate distribution. While initially developed for Poisson-like spike activity,^220^ this approach has been later found to be inadequate for many neuronal systems.^221^ More recent methods have adopted alternative strategies that rely on statistical assumptions that better reflect the characteristics of real spike trains.^222,223^

Beyond these two main categories, studies have explored hidden Markov models^224^ and machine learning-based approaches^241^ for automated burst detection. However, while some methods have shown improved overall performance in specific contexts, no method has proven to generalize well across different experimental conditions without manual parameter adjustments.^213^

**Fig. 10:**
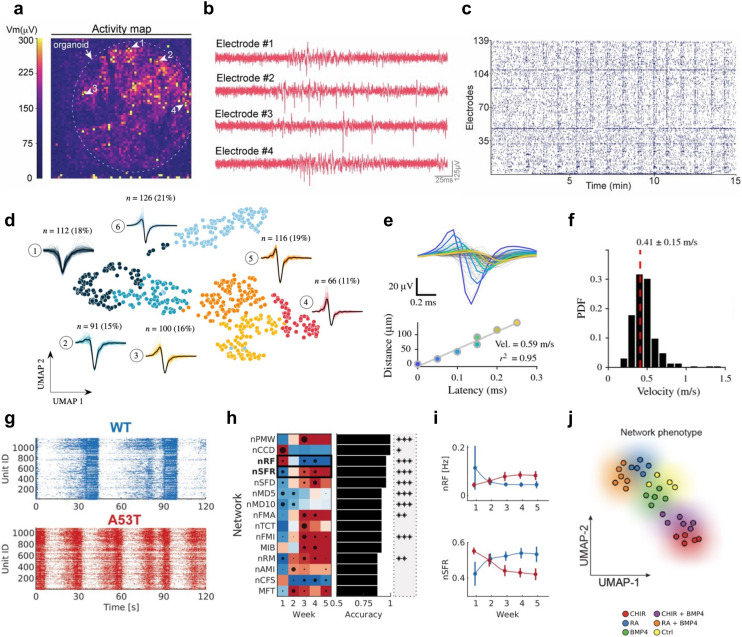
Functional characterization of stem cell-derived human neurons and disease modelling with high-density microelectrode arrays. a–c, Spontaneous electrical activity recorded from human brain organoids placed on an HD-MEA. a, Whole-array activity map of a 4-months-old organoid. b, Four example traces of active electrodes on the HD-MEA (see arrows in panel a). c, Raster plot of an HD-MEA organoid recording over 15 min, showing some network bursts. d, UMAP (Uniform Manifold Approximation and Projection) plot of AP waveforms from neurons in 3–4-months-old human brain organoids recorded on an HD-MEA; each dot indicates a single unit. The coloring indicates six different clusters obtained by a Louvain modularity analysis; the corresponding peak waveform of each unit and cluster are depicted in matching colors. e and f, Estimates of axonal AP velocity in neurons from human brain organoids. e, Electrical footprint (EF) of a single unit; the signals are color-coded according to their latency, starting from the electrode featuring the strongest negative signal amplitude (see top panel). AP propagation distance *versus* latency plot for this unit, along a linear fit to estimate the axonal conduction velocity. f, Histogram of axonal AP velocity estimates across the recorded organoids. g, Raster plots of spontaneous activity from two human iPSC-derived dopaminergic neuron cultures recorded on HD-MEAs. The upper panel shows the activity recorded from healthy (WT) dopaminergic neurons; the lower plot shows the activity of dopaminergic neurons with the A53T point mutation, which has been associated with familial autosomal Parkinson's disease. h, Heatmap showing differences in network-level features between WT and A53T dopaminergic neurons over five weeks *in vitro*. Black dots within each panel represent the relative predictor importance at each time point. The horizontal black bars in each row indicate the accuracy of a random forest classifier in distinguishing between both cell lines, and asterisks denote the significance levels based on linear mixed models. i, Development of two example network features tracked over five weeks (A53T in red, WT in blue). j, UMAP clustering plot of induced human glutamatergic neurons, patterned with five different morphogen combinations. Each dot corresponds to one culture, with colors indicating the different patterning conditions. The results demonstrate that morphogen patterning gives rise to functionally diverse neuronal subtypes, which can be distinguished based on HD-MEA-derived network features. Panels a–c were reproduced with permission from ref. 229; copyright (2024): Springer Nature Publishing. Panels d–f were reproduced with permission from ref. 5; copyright (2024): the authors, reproduced under the CC BY 4.0 license; panels g–i were reproduced with permission from ref. 4; copyright (2023): the authors, reproduced under the CC BY 4.0 license. Panel j was adapted with permission from ref. 230; copyright (2025): the authors, reproduced under the CC-BY 4.0 license.

HD-MEAs enable advanced analyses of the spatiotemporal profiles of NBs that go beyond the coarse-grained quantifications possible with standard MEAs. The higher resolution allows for reconstructing burst propagation across the entire network,^226^ and provides a better characterization of burst shapes and similarities in their spatiotemporal profiles.^227^ Such burst shape features have been used to differentiate between cell lines and to assess drug effects on neuronal networks.^4,6^ For example, Ronchi *et al.*^6^ demonstrated that the shape and occurrence patterns of NBs in human iPSC-derived neurons are highly indicative of the respective cell lines, enabling a clear distinction between motor and dopaminergic neuron networks. Similarly, Hornauer *et al.*^4^ demonstrated that burst-related measures could distinguish healthy from diseased dopaminergic neuron cultures and predict their age and treatment conditions (Fig. 10g–i). Finally, studies probing the initiation mechanisms of NBs, combined optogenetic stimulation with HD-MEA recordings and demonstrated that NBs can be triggered by activating so-called “leader neurons”.^97^

Maccione *et al.*^231^ used HD-MEA recordings to track the spatiotemporal profiles and network dynamics in retina explants. Analyzing retinal waves of neonatal mice, they quantified the wave size, area, speed as well as the recruitment of specific cell types. The presence of waves was detected using a two-step burst algorithm,^232^ and their propagation was further characterized using an algorithm that estimated center-of-activity trajectories (CATs).^226^ Analyzing the spatiotemporal profiles of retinal waves from birth until eye-opening, the authors found that early cholinergic retinal waves (postnatal day (P) 2–6, stage II waves) were large and showed more random trajectories. After P7, when GABA becomes inhibitory in the mouse retina, the waves became smaller and slower. From P10 onwards (stage III), glutamate-sensitive waves exhibited more repetitive, faster and spatially confined trajectories.

Finally, several studies have looked for signs of self-organized criticality (SoC) in the spontaneous activity of neuronal networks.^233,235^ SoC has been a popular concept to describe the emergence of complexity in various natural systems, where temporal and spatial cascades of events follow scale-free power law distributions.^234^ Recordings obtained from organotypic cortical slice cultures using high-count MEAs (512 channels, 60 μm electrode pitch) revealed propagating neuronal activity cascades – termed “neuronal avalanches” – that exhibited hallmarks of critical dynamics: the size distribution of neuronal avalanches followed a power law, there was an avalanche shape collapse, and the relationship between avalanche size and duration was consistent with criticality.^236^ In dissociated neuronal cultures, studies fitted power laws to the size distribution of spatiotemporal activity patterns and revealed a developmental trajectory toward SoC.^237^ Neuronal networks transitioned from a low-activity, Poisson-like state (at day *in vitro* (DIV) 4) to activity patterns near criticality at later stages (DIV 16). Although the progression toward SoC has also been reported in earlier studies using low-density MEAs,^235^ the developmental timing of this dynamical state varies between studies. It is noteworthy that both studies based their avalanche statistics on MUA rather than spike-sorted data.

#### Connectivity inference

Inferring connectivity from large-scale neural recordings remains a pivotal step in understanding neural network development and the putative functional role of specific neurons.^238^ While different concepts to describe neuronal connectivity exist,^239^ structural connectivity traditionally refers to the physical wiring of the network at the cellular level – that is, the presence or absence of synaptic connections. Functional connectivity refers to the statistical dependencies or correlations between neuronal spike trains, and effective connectivity represents the directional influence that one neuron exerts on another neuron, implying – for some methods – a causal relationship (*cf.* Box 4).^240^

**Box 4: Algorithms for functional connectivity inference**

Inferring connectivity from HD-MEA data is a powerful approach to uncovering the functional architecture of neuronal networks. HD-MEA recordings primarily enable the inference of functional and effective connectivity. Although these methods do not directly reveal structural connections between neurons, they can help to infer patterns of interaction and information flow based on observed spike trains and their temporal relationships. The most common approaches for inferring connectivity from HD-MEA data can be broadly grouped into **statistical**, **model-based**, and **information theory-based** methods (see ref. 240 and 242 for recent reviews).

**Statistical methods** often rely on cross-correlation or covariance measures between spike trains to infer connections, based on the assumption that correlated activity between neurons may indicate a potential synaptic connection (*cf.* ref. 243–245). A more sophisticated statistical approach is Granger causality, which assesses whether the past activity of one neuron can be used to predict another neuron's future activity, thus implying a directional influence.^246^

**Model-based approaches** include Generalized Linear Models (GLMs), which describe the spiking activity of a neuron as a function of the activity of other neurons, incorporating external covariates and taking into account the temporal structure of the data.^247,248^ Recent studies have combined statistical and model-based approaches, for example by decomposing cross-correlograms into multiple components,^249–252^ or by applying post-processing strategies to refine connectivity estimates.^253,254^

**Information theory-based methods** leverage metrics, such as mutual information, to quantify the amount of shared information between spike trains.^240^ Unlike correlation-based methods, these approaches do not assume linear relationships and are able to detect more complex statistical dependencies. Transfer entropy (TE), an extension of mutual information, is particularly useful for detecting directed interactions between neurons. It quantifies how much the past activity of one neuron improves the prediction of another neuron's future activity beyond what can be explained by the latter's own history.^255,256^

Studies have inferred connectivity from HD-MEA recordings across a variety of preparations, including acute brain slices,^29,105,106^ organotypic slices,^257–259^ and rodent or human neuronal cultures.^49,260^ While most of these studies focused on functional and effective connectivity, parallel HD-MEA/patch-clamp recordings have enabled the reconstruction of cell-type specific synaptic connectivity within smaller subsets of neurons (*cf.* section 3.2; Fig. 8).^99,100^ Analysis code for inferring synaptic connectivity from such combined recordings is publicly available.^100,261^

Beyond the type of neuronal preparation, the interpretability and biological relevance of the inferred connectivity depends critically on the quality of the input data. Accurate estimation of synaptic connectivity between single units requires spike-sorted, quality-controlled data with sufficient spiking activity to capture short-latency interactions, indicative of mono-synaptic connections.^240^ Strongly recurrent network activity (*e.g.*, including many NB), incomplete sampling, or suboptimal experimental conditions may alter connectivity inference and lead to false/incomplete estimates.^262^ Future work should further examine how direct or indirect electrical coupling between neurons, such as *via* gap junctions, affects the fidelity of activity-based connectivity estimates.^263^

A widely applied method to estimate functional or effective connectivity from HD-MEA recordings is transfer entropy (TE).^257–259,264,265^ Multivariate extensions of TE have been developed to infer connectivity from spike trains and proven robust even in challenging inference scenarios involving highly correlated activity or common drive.^255,257^ Studies have also started to use artificial neural networks (ANNs) for connectivity inference, either trained on synthetic data^266^ or the output of existing inference methods.^261^ Donner *et al.*,^261^ for example, introduced an ensemble ANN approach that outperformed several conventional inference algorithms and provided insights into the specific features that most influenced network reconstruction performance.

Finally, the ability of HD-MEAs to track both individual neurons and network dynamics over extended periods provides a unique opportunity to study the generative wiring rules underlying neuronal network formation *in vitro*.^260^ By linking developmental changes in neuronal activity patterns to evolving connectivity, HD-MEAs enable researchers to investigate how networks self-organize over time.^238^ As such, HD-MEAs represent a powerful platform for unravelling the principles of neuronal communication, the emergence of network topology, and the temporal evolution of information flow in neural systems.

#### Extracting information from AP waveforms

Beyond spike-train statistics, it is well established that AP waveform features provide valuable information about cell classes (*e.g.*, excitatory *versus* inhibitory neurons),^267^ their anatomical location within the brain,^268^ and neuronal physiology (Fig. 1). As demonstrated by combined intra- and extracellular recordings, the extracellular AP shape resembles the first temporal derivative of the intracellular AP, and arises from transmembrane currents – in particular the inward sodium current mediated by voltage-gated sodium channels.^159,269,270^ The extracellular AP waveform and its polarity vary depending on the electrode’s position relative to the neuronal compartment (*e.g.*, dendrite, soma, axon initial segment, or axon), as well as on the neuron's morphology and the local microenvironment.^270–272^ Moreover, as an indirect representation of ion channel expression and dynamics, differences in AP waveform features can be indicative of pathological alterations – such as those caused by ion channel mutations implicated in epilepsy. While patch-clamp techniques have been most commonly used in this context, extracellular features have been shown to reproduce some features observed in intracellular recordings.^273^

In order to enable a systematic comparisons, AP waveforms have typically been quantified through specific features, such as the AP duration or half-width (*e.g.*, to distinguish “broad” from “narrow” spiking neurons) or the trough-to-peak delay.^274,275^ However, a significant overlap in some basic waveform features, such as AP widths, has also been reported when comparing different cell classes, at least in some species. More recent approaches have therefore used the entire spike waveform as input for dimensionality reduction techniques, such as UMAP (Uniform Manifold Approximation and Projection), which have demonstrated improved sensitivity in detecting subtle differences in spike waveform shapes.^225^ While initial work demonstrated the potential of this approach *in vivo*, similar methods have been applied to monolayer cultures and human brain organoid slice recordings (Fig. 10d).^4,5,272^ Future research and ground-truth datasets are needed to determine how and when AP waveform classification schemes developed for *in vivo* use can be translated to HD-MEA *in vitro/ex vivo* studies,^276^ and which alternative waveform features could improve classification performance.^100^ An interesting direction in this context was proposed by Shein-Idelson *et al.*,^122^ who introduced the concept of so-called “spike-induced fields” (SIFs) and demonstrated that these spike-triggered extracellular waveforms contain synaptic signals indicative of neuronal identity, enabling their use in cell-type classification analyses (Fig. 9).

Several studies have investigated the extracellular and intracellular-like AP waveform features of cardiomyocytes (CMs) using HD-MEAs, employing chips with dedicated microstructures (*cf.* section 2.4) or modified electrode surfaces (*e.g.*, electrodeposition of dendritic Pt-black nanostructures, Fig. 5e and f).^34,35,277,278^ Following an electro- or optoporation step to open the CM membrane, these studies demonstrated that specific features of the intracellular-like AP waveform changed in a dose-dependent manner in response to various ion channel modulators. Moreover, Rahmani *et al.*^279^ showed that extracellular activity of CMs recorded on nanoelectrode arrays could be used to train deep learning models to predict intracellular AP waveform features from extracellular time series. These results suggest that micro- and nanoelectrode devices represent a promising tool for high-throughput cardiac safety drug screening, enabling simultaneous access to extracellular and intracellular-like AP signals.

#### Reconstructing electrical footprints and axonal propagation dynamics

The dense arrangement of electrodes on HD-MEAs enables electrical imaging at subcellular resolution. The typical workflow to obtain such high-resolution data begins with whole-array high-density recordings, performed either sequentially on chips with a SM readout scheme or simultaneously on APS-based^280^ or dual-mode chips.^8,49^ This step is followed by spike sorting of the data and the subsequent inference of electrical footprints (EFs) of individual neurons using spike-triggered averaging. An EF represents the average extracellular electrical potential distribution of a neuron on the HD-MEA (Fig. 1a, c and f; Fig. 4a). Depending on the electrode pitch of the HD-MEA, the spatiotemporal information captured by the EF can be highly detailed and may allow for the identification of subcellular compartments.^24,272^

Several studies have used HD-MEAs for functional electrical imaging of neurons, and have tracked AP signals along individual axonal branches.^102–104,230,281,282^ Spike-triggered averaging during periods of low population activity allows even small-amplitude signals to be isolated from the noisy background.^102,103,282^ The resulting whole-array EFs have been employed to trace axonal arbors, providing insights into neuronal morphology and AP propagation features (*e.g.*, conduction velocity and branch point failures) that cannot be captured with conventional imaging methods.^8,104,281^

To track the dynamics of individual AP propagation, innovative template-matching algorithms have been introduced.^103^ While mapping these reconstructions to ground-truth imaging data remains challenging, this approach has proven to be sensitive enough to differentiate conduction dynamics across different neuron types.^104^ Finally, studies also demonstrated, that incorporating spatial EF information in the form of multichannel features can improve cell type classification performance.^230,268^

#### Long-term tracking of single-cells and networks

A key advantage of HD-MEAs is their ability to record from single neurons and neuronal networks at very high temporal resolution (on the order of milliseconds) over extended time periods – ranging from days to months.^5,206,260^ This offers a clear benefit over optical imaging, which, while capable of parallel recordings from a large number of neurons, is limited by phototoxicity effects during long-term experiments. HD-MEA-based developmental staging provides valuable insights into biologically relevant single-cell properties over time, such as changes in AP waveform features, single-unit firing statistics, and axonal morphology (Fig. 1). Furthermore, with carefully selected recording configurations, HD-MEAs can be used to track the maturation and self-organization of neuronal networks as development progresses.^260^

The analysis of long-term developmental data has been greatly facilitated by increasingly robust spike-sorting pipelines, which can handle large, concatenated datasets.^92,283^ Moreover, there is now custom software available to track individual units across multiple spike-sorted recordings.^206^ While studies demonstrated the feasibility to follow neurons in rodent primary networks^260^ and human organoid slice cultures^5^ across days *in vitro*, it is important to note that studies have also reported considerable movement of cells on HD-MEAs.^228^ Such movement can depend on various factors, including the cell type, the maturity and health of the neurons, and the type of coating used to plate cells on the HD-MEA surface. Although many spike-sorting algorithms can cope with some degree of spatial displacement, the tracking yield of cells will likely be higher, if these parameters are considered during experimental planning.

### 4.3. Advancing neuronal interfaces with HD-MEAs

HD-MEAs allow for simultaneous recording and stimulation of several tens to hundreds of neurons,^70,71^ making them ideally suited for bidirectional interfacing between biological tissue and off-chip computational devices. Integrated into closed-loop systems, HD-MEAs hold significant promise for advancing brain–machine interfaces (BMIs) and neuroprosthetic technologies, as well as for deepening our understanding of neuronal coding more generally. While lower-density MEAs can be used to control firing rates at the network level,^284^ more advanced experimental paradigms – such as the study of synaptic plasticity – require the single-cell resolution provided by HD-MEAs.^285^

In recent years, the high spatiotemporal resolution of HD-MEAs has been leveraged to expose *in vitro* neuronal networks to simulated virtual environments as a means to probe learning behavior.^286^ For example, studies have reported that human and mouse neurons improved their performance in response to the feedback provided by a closed-loop system while playing the game ‘Pong’.^286^ A follow-up study reported that these neuronal networks operated in a near-critical state when receiving task-relevant input.^287^ Seemingly, these HD-MEA-interfaced biological neural networks even outperformed state-of-the-art reinforcement learning algorithms, suggesting superior computational efficiency.^288^ This putative computational efficiency of neurons was further investigated in a recent study that utilized the plasticity of brain organoids to process spatiotemporal information within an adaptive reservoir computing framework.^289^ In this work, organoids were stimulated and recorded on HD-MEAs, serving as living biological reservoirs, and changes in functional connectivity were observed during an open-loop learning experiment. Finally, in a closed-loop real-time biohybrid experiment, HD-MEAs and organoids were integrated with a biomimetic spiking neural network, enabling interaction between real and artificial neurons.^290^ The authors proposed that the introduced platform could support the development of future neuromorphic-based prostheses.^290^

Some of the mentioned studies have been met with skepticism by the community, and concerns were raised about the adequacy of experimental controls and the use of terms like sentience, intelligence and computation when referring to *in vitro* cultures. These critiques underscore the need for more precise and context-appropriate terminology, as well as clearer reporting standards in the field.^291^ Such refinements would enhance the reproducibility of future studies and help clarify the boundaries of what these advanced systems can – and cannot – contribute to our understanding of the biological systems under investigation.

## 5. Challenges

Although HD-MEAs offer unique capabilities for the characterization of electrogenic cells, as reviewed in the previous sections, they also present important challenges and limitations that have to be addressed. The relevance of these challenges depends on the specific research question and application. In this section, we abstract our current understanding of the biocompatibility of HD-MEAs and discuss how their materials and surface properties may affect cellular physiology. We also discuss some practical challenges that relate to the operational performance of HD-MEAs, including challenges associated with their opacity and light sensitivity, electrical stimulation, and more general issues such as data readout, data sharing, and the need for standardized analysis pipelines.

### 5.1. Biocompatibility

The choice of materials used for fabricating HD-MEAs, especially at the biointerface, is critical to ensure that these devices do not only feature good recording performance, but also sufficient biocompatibility. While biocompatibility is equally important for *in vitro* and *in vivo* applications, most studies so far have focused on the stability and compatibility of neuronal interfaces in living animals. For robust functional readouts from *in vitro* cellular systems – such as neuronal cultures derived from human iPSCs, which require extended maturation periods – rigorous biocompatibility and stability testing is essential. This includes comprehensive evaluations across diverse cell types to ensure that HD-MEA technologies are broadly applicable, reliable, and do not interfere with normal cellular function.

#### Microelectrode materials

Materials such as indium tin oxide (ITO) and titanium nitride (TiN) have been extensively utilized for realizing thin-film electrodes of MEA systems, due to their high electrical conductivity and optical transparency.^292,293^ However, electrodes in HD-MEA systems are predominantly made from biocompatible metals like platinum (Pt), iridium, and gold. Because HD-MEA electrodes are very small, they typically have high impedance and limited charge injection capacity.^294,295^ To address these issues, dendritic materials, such as Pt black – which increases the surface area of electrodes – have been applied to lower electrode impedance and improve signal transduction.^294,295^ Yet, Pt black also has drawbacks, including its limited lifespan, brittle nature, and potential cytotoxicity.^296,297^

To overcome some of these limitations, alternative electrode materials, such as electropolymerized conductive polymers have been used.^298^ Among them is poly(3,4-ethylenedioxythiophene) doped with poly(styrene sulfonate) (PEDOT:PSS), which enhances electrode performance, supports cellular viability, and enables reproducible recordings.^299–301^ However, an important limitation of such organic materials is their instability, which can make stable long-term recordings challenging.^302,303^

In summary, while there are advances in developing stable and biocompatible electrode materials, further improvements are needed to enhance electrode performance and long-term stability. Damaged electrodes or faulty connections can increase noise levels and degrade overall recording quality, which in turn can impair the detection of neuronal activity and affect downstream data processing steps such as spike sorting.

#### Extracellular matrix and mechanical properties of HD-MEAs

It is well established that both the mechanical properties of the cellular environment and the chemical composition of the extracellular matrix (ECM) influence the differentiation potential of cells,^304^ their morphology, and even the activity of neuronal^305^ and cardiac cells.^306^ Taking these findings into consideration is especially important for studies that use HD-MEAs chronically, for example, to track cellular development over extended time periods, or to study functional phenotypes. To address potential adverse effects caused by the mechanical properties or surface chemistry of HD-MEAs, several studies have explored the use of specific ECM-like coatings to enhance cell adhesion and viability. While functionalizing HD-MEA surfaces and application of specific biomolecules (*e.g.*, laminin) can likely mitigate some of these effects,^184^ future research is needed to systematically study how the mechanical properties of HD-MEAs alter the physiology of cells. This also holds true for more recently introduced 3D HD-MEAs, which incorporate nanoscale structures to penetrate tissue and/or cells on demand (*cf.* section 2.4). An innovative approach to circumvent potential alterations introduced by stiff surface properties was recently presented by Han and colleagues.^307^ In this study, human neurons were cultured on a device composed of electrospun polystyrene (ESPS) fibers, which were subsequently transferred onto HD-MEAs for electrophysiological recordings. The authors found that long-term culturing on the fiber device was feasible, suggesting that their approach may be suitable for improving the microenvironment of *in vitro* neurons without compromising their compatibility with HD-MEA platforms.

### 5.2. Operational challenges

#### Opacity and light-sensitivity of HD-MEAs

A key limitation of CMOS-based HD-MEAs is the opacity of their sensing areas, which complicates simultaneous live-imaging, immunohistochemical studies, and optogenetic experiments – unless specific upright microscopes are available.^101^ To address this, several mitigation strategies have been proposed. For example, studies have explored the use of flexible microelectrode sheets to enhance optical transparency and improve access for microscopy.^95^ While these interfaces feature fewer and less densely packed electrodes, they could represent a promising solution for studies that rely on optical means.

Another operational challenge is the light sensitivity of HD-MEAs. The light intensity levels required for activating optogenetic constructs range from tens to hundreds of mW cm^−2^, whereas stimulation of, *e.g.*, retinal photoreceptors requires only intensities of tens of μW cm^−2^. Exposing light-sensitive components of HD-MEAs, such as on-chip transistors, to strong light stimuli can introduce light-induced artifacts, increasing noise levels or generating erroneous signals in the recordings. Notably, MEAs and HD-MEAs with their sensitive units and circuits positioned away from the electrode area are less susceptible to light-induced artifacts.^37^ Such chips mostly rely on SM architectures that minimize the placement of sensitive units directly underneath the electrode array. In contrast, HD-MEAs that incorporate in-pixel amplifiers and circuitry are more prone to light-induced artifacts, even when the light is focused only on the electrode array. Nevertheless, the studies discussed here demonstrate that careful experimental design and targeted modifications of the HD-MEA platform can reduce the severity of light-induced artifacts.^26,112,113,308^ In addition to hardware-based solutions, alternative mitigation strategies, such as spike sorting and artifact removal, have been developed.^309^

#### Artifacts from electrical stimulation

An additional operational challenge is the occurrence of stimulation artifacts resulting from electrical stimulation on HD-MEAs (*cf.* section 2.2). Usually, mV-range signals are used for stimulation, while μV-range signals are read out at the same time. Applying large stimulation signals to a specific electrode can induce significant voltage fluctuations in the surrounding medium, which are then picked up by nearby electrodes. This effect becomes particularly problematic when the artifacts are large enough to saturate the recording amplifiers, as it can prevent the circuits of nearby electrodes from detecting signals for tens of milliseconds or longer.

To address this issue, it is crucial to carefully design a stimulation protocol that effectively stimulates the target cells, while minimizing artifacts. One method for suppressing stimulation artifacts is the use of blanking readout channels.^310^ This technique involves temporarily disabling the readout channels during electrical stimulation to prevent saturation through high-amplitude stimulation signals or artifacts generated by the pulses. Once the stimulation is completed, the readout channels are re-enabled to resume normal signal recording. This method shields the readout circuits from the large stimulation signals that could otherwise lead to long-lasting saturation. However, the process of blanking the readout channels itself can introduce unwanted artifacts and may require recovery time before normal signal detection resumes.^311^

Alternatively, readout circuits can incorporate mitigation methods. For example, Shadmani *et al.*^67^ integrated both voltage and current stimulation in their system, and their current stimulation pathway included a voltage limiter that restricted the current if aberrant/unsafe voltage levels were detected. They also introduced a soft-reset mechanism in the voltage recording channels, which dynamically adjusted the amplifier frequency response by pole shifting (*i.e.*, shifting to higher frequencies and lower amplification for the stimulation duration). This enabled rapid amplifier recovery from saturation – typically within 200 μs, which is shorter than the ∼2 ms duration of an AP. Another study introduced a CMOS-based MEA featuring digitally assisted, closed-loop charge balancing circuits that prevented residual charges in the sample.^41^ In addition to advances on the hardware side, studies have also proposed new algorithmic solutions, such as a structured Gaussian process model, to model and account for electrical stimulation artifacts in HD-MEA recordings.^312^

#### Precision of electrical stimulation

Achieving single-cell resolution electrical stimulation is one of the key advantages of HD-MEAs. However, to precisely stimulate individual neurons presents several challenges.^9,10,102^ First, neurons are rarely perfectly aligned with a single electrode. Depending on the sample and its positioning, stimulating a specific neuron may be difficult – or even impossible. Another challenge is determining a suitable stimulation amplitude. If the stimulation is too strong, it can activate multiple neurons simultaneously or, sometimes, even cause damage to the cells and the electrodes. Conversely, if the stimulation is too weak, it may fail to evoke any response. The optimal stimulation amplitude is highly dependent on the electrode's position relative to the neuron. For instance, electrodes positioned directly beneath the AIS typically require lower amplitudes than those near the dendrites or soma.^24^ Ideally, the stimulation parameters should be adapted and calibrated for each specific experimental setup. However, this calibration process is complicated by stimulation artifacts, which may obscure recordings immediately after stimulation and make it difficult to determine whether an AP in the targeted neuron has been successfully evoked. Addressing these challenges is crucial for leveraging the full potential of HD-MEAs.

#### Interfacing neuronal tissue to planar HD-MEAs

Most currently available HD-MEAs feature a planar sensing area and excel at recordings of electrogenic cells and ensembles that are in direct contact with or in close proximity to the microelectrodes. However, planar recording surfaces can present challenges when tissues do not naturally conform to the array. Despite these limitations, an increasing number of studies have successfully used HD-MEAs to record from tissues such as retinae or acute brain slices obtained from various animal models and humans.^98,112,313^ In most studies, the samples were immobilized on the array using tissue harps or more elaborate holder structures that gently press the tissue onto the array. While these approaches enable robust recordings, further research is needed to assess how mechanical stabilization methods influence the recorded electrophysiological activity.

Alternative approaches to planar arrays are HD-MEAs with 3D micropillars^96^ or massively parallel microwire-bundles connected to CMOS arrays (*cf.* section 2.4).^87,95^ While these devices can penetrate tissue and compensate for spatial mismatches, more research is needed to understand how they affect the functionality and activity of the studied tissue.

Another promising alternative to rigid HD-MEAs involves devices with flexible or stretchable electronics.^175^ These conforming recording devices reduce the mechanical mismatch between tissue and device and can adapt to developmental or morphological changes. This renders them particularly suitable for interfacing with mechanically active biological tissue, such as cardiac or neuromuscular organoids.^314^

#### Data readout and processing challenges

A critical challenge in HD-MEA studies is the substantial volume of data generated. A typical readout channel at ∼20 kSps with 10-bit resolution produces about 200 kbps. As the number of channels amounts to several thousands, the total data rate can surpass several gigabits per second, presenting significant challenges for data transmission, storage, and post-processing. There are fundamentally two approaches to address this: one is to transmit and store all captured data off-chip; the other is to perform on-chip selection to discard irrelevant data before transmission.

The first approach – continuous data storage – preserves the full raw signal, providing greater flexibility for post hoc analysis. Retaining raw signals enables researchers to apply a range of algorithms and signal processing methods to determine the most effective approach, and to retrospectively tailor these methods to the dataset. Additionally, advanced filtering techniques can be employed to eliminate potential interferences, such as 50/60 Hz power-line noise. However, this approach requires high-performance data acquisition infrastructure capable of rapid data transfer and storage, such as solid-state drives (SSDs) or USB3 interfaces. Most HD-MEA systems store raw data in HDF5 format, a widely adopted standard for large-scale scientific datasets.

The second approach involves on-chip processing to limit the amount of data that needs to be transferred and stored. An example for this method is event-triggered acquisition, in which data are saved only when APs are detected. More advanced strategies involve compressing or filtering the data directly at the source. For instance, Tsai *et al.*^43^ proposed the use of compressive-sensing concepts, although severe issues were later identified with this approach.^315^ Jang *et al.*^46^ introduced a strategy that reduced data volume by combining PPM-based ADPs with wired or lossy compression. On-demand lossy compression schemes and signal masking have also been implemented in commercially APS-based HD-MEAs to reduce data rates. Finally, Cartiglia *et al.*^48^ designed an asynchronous event-based HD-MEA that outputs data only when electrode voltages change, which further reduced data volume.^48^

## 6. Conclusion and outlook

In this *Tutorial Review*, we have summarized recent advancements in HD-MEA technology and its application across a wide range of biological systems over the past decade. We began by introducing critical technological innovations that have established HD-MEAs as attractive platforms for large-scale electrophysiology and significantly advanced their capabilities. We then surveyed the wide range of experimental settings in which HD-MEAs have been applied, with a focus on *in vitro* and *ex vivo* studies. Many of these applications benefitted from combinations with complementary techniques to obtain robust physiological insights.

Equally important as the technological advancements are innovations in algorithms and analysis techniques for handling the large and complex datasets generated by HD-MEAs. We thus highlighted essential computation routines to infer electrophysiological features at various biological levels – some of which are difficult, if not impossible, to obtain with optical or other electrophysiological methods. Finally, we discussed key challenges of current HD-MEA systems and outlined some potential mitigation strategies.

As HD-MEA technology has matured, an increasing number of studies have demonstrated its potential for high-resolution functional characterization of electrogenic cells. As reviewed, HD-MEAs feature highly precise recording and stimulation capabilities across spatial and temporal scales, providing valuable insights into fundamental electrophysiological phenomena – ranging from the study of cell-type specific neuronal connectivity and synaptic plasticity to axon conduction properties and the excitability of neuronal compartments. Access to these biological features offers significant promise, not only for basic research, but also for translational applications, such as the functional characterization of human cellular disease models and drug screenings. Although HD-MEAs generate large amounts of data, advances in data analysis techniques increasingly enable efficient extraction of biologically meaningful electrophysiological features, which can complement other cellular readouts, including transcriptomics and proteomics.^316,317^

We anticipate that the growing commercial availability of *in vitro* HD-MEAs – offered by companies, such as MaxWell Biosystems (http://www.mxwbio.com), 3Brain (http://www.3brain.com), CytoTronics (http://www.cytotronics.com), and Multi Channel Systems (http://www.multichannelsystems.com) – will significantly accelerate their adoption and contribute to the refinement of this technology in the years to come. While custom-built or non-commercial HD-MEAs feature full flexibility in chip design and application, today's commercial systems already offer a broad range of features that allow customization by the user (*cf.* section 3). Available formats include both single-well and multi-well HD-MEAs. Single-well systems are well suited for advanced recording and stimulation experiments and can be readily combined with complementary readouts. In contrast, multi-well formats (*e.g.*, 6- and 24-well plates) enable parallel experimentation and are particularly advantageous for higher-throughput applications, such as compound screening. Many systems are equipped with user-friendly software and modular assays that can be tailored to specific experimental questions. Some platforms even support control *via* Application Programming Interfaces (APIs), which facilitates seamless integration into other workflows and ensures compatibility with lab automation. The studies covered here underscore that HD-MEAs have emerged as a highly versatile platform technology, which is now deployed to a growing number of laboratories across diverse scientific disciplines.

Although this review has focussed on advances in HD-MEA technology and its applications to neuronal and cardiac cells *in vitro*, there are numerous other electrogenic cell types and tissues that could be further investigated using these devices. For example, HD-MEAs have been used to study the differentiation of primary skeletal muscle cells into electrically active myotubes,^318^ as well as signal transmission at the neuromuscular junction.^149^ Other work has examined the electrophysiological properties of endocrine/neuroendocrine cells, including pancreatic islets,^319^ and chromaffin cells.^320^ HD-MEAs have also been employed for impedance-based measurements to characterize dynamic processes in non-excitable cells *in vitro* – for example, to study epithelial barrier formation^321^ or to investigate the behavior of various cancer cell types.^30^ Finally, future studies could leverage the capabilities of *in vitro* HD-MEAs for advanced functional monitoring in complex microphysiological systems, including models of the blood–brain barrier and vascularized tissues.^322^

In addition to advancements in HD-MEAs for *in vitro* applications, significant efforts are underway to develop high-density microelectrode probes for *in vivo* use in humans, with several devices currently undergoing pre-clinical and clinical testing. To date, however, only one traditional multi-channel device – the NeuroPort Array by Blackrock Neurotech (http://www.blackrockneurotech.com) – has received approval by the U.S. Food and Drug Administration (FDA) for use in humans. The NeuroPort Array consists of a 10 × 10 grid of ∼1 mm long electrodes at 400 μm electrode pitch, distributed over a 4 × 4 mm^2^ area. Numerous companies are developing brain–machine interface (BMI) or brain-computer interface (BCI) implants that leverage CMOS-based fabrication techniques to expand on the capabilities of such existing devices. This development is both timely and encouraging, since our understanding of the limitations and side effects of existing neuromodulatory devices on brain tissue has grown.^78^

Among the companies that are racing to bring the next-generation of BCIs to the market is Neuralink (http://www.neuralink.com), which began testing of its N1 implant in patients with quadriplegia in 2024. The N1 is a flexible device containing 1024 microelectrodes, distributed across 64 ultra-thin polymer threads, each of which accommodates 16 electrodes. In the same year, Paradromics (http://www.paradromics.com) started safety testing of its Connexus BCI, a modular system containing up to four units, each equipped with 421 microwire electrodes that penetrate 1.5 mm into the cortex. Another startup is Precision Neuroscience (http://www.precisionneuro.io), which is currently evaluating its layer 7 cortical interface, a scalable micro-electrocorticography (μECoG) device featuring 1024 microelectrodes on a ∼1.5 cm^2^ flexible polyimide film. Innovative BCI research is also conducted by startups like Corticale (http://www.corticale.com), which is marketing a modular, CMOS-based high-density BCI system featuring 1024 electrodes per shank at 30 μm pitch, along with wireless transmission.^84^ Finally, there are published intraoperative recordings using the Neuropixels system (http://www.neuropixels.org). The Neuropixels probes are developed by IMEC in Belgium (http://www.imec-int.com), and proof-of-concept that these devices can be used for large-scale single-unit recordings in humans has been provided.^323–325^

What will define the next generation of HD-MEAs – and what will they look like? While there may be technological limitations concerning the number of microelectrodes that can be realized in HD-MEAs, or the overall sensing area of these devices, we are likely to see a diversification of devices designed for specific research fields or tissues of interest. As discussed, developments towards more specialized HD-MEAs are already underway with the adaptation of existing systems for 3D extracellular measurements, large-scale intracellular recordings, and adaptations for *in vivo* applications. Since most neuronal tissues are not monolayers, improving 3D electrical imaging will be particularly important. However, whether such measurements will be possible – and necessary – at a spatiotemporal resolution currently achievable in 2D remains to be seen. As always, there will be a trade-off between the technical feasibility to sample specific parameters and the information that will be required to answer biologically relevant scientific questions.

## Conflicts of interest

There are no conflicts to declare.

## Data Availability

No primary research results, software or code have been included and no new data were generated or analysed as part of this review.
